# Effects of Swapping 5HT3 and α7 Residues in Chimeric Receptor Proteins on RIC3 and NACHO Chaperone Actions

**DOI:** 10.3390/molecules30214235

**Published:** 2025-10-30

**Authors:** Zixuan Yan, Sreeharshini Oruganti, Swetha K. Iyer, Kushboo Agarwal, Mitali Gupta, Ketaki Adhikari, Nevetha Vijayan, Jash Doda, Vaishali Jain, Arya N. Lokhande, Aadhya P. Nair, Venkat Sri K. Nallanichakravarthula, Maegan M. Weltzin, Ralph H. Loring

**Affiliations:** 1 Department of Pharmaceutical Sciences, Northeastern University, Boston, MA 02115, USA; yan.zixua@northeastern.edu (Z.Y.); oruganti.s@northeastern.edu (S.O.); kshwetha95@gmail.com (S.K.I.); agarwal.ku@northeastern.edu (K.A.); gupta.mi@northeastern.edu (M.G.); adhikari.ke@northeastern.edu (K.A.); vijayan.ne@northeastern.edu (N.V.); doda.jas@northeastern.edu (J.D.); jain.vaish@northeastern.edu (V.J.); lokhande.ar@northeastern.edu (A.N.L.); nair.aad@northeastern.edu (A.P.N.); nallanichakravarth.v@northeastern.edu (V.S.K.N.); 2 Department of Chemistry and Biochemistry, Institute of Arctic Biology, University of Alaska Fairbanks, Fairbanks, AK 99775, USA; mmweltzin@alaska.edu

**Keywords:** alpha-bungarotoxin binding, chaperone actions, transmembrane domains, receptor subunit folding, receptor assembly, quantitative fluorescent binding assay, ImageJ analysis

## Abstract

Alpha7 nicotinic receptors (α7-nAChRs) are implicated in many neurological disorders, but how they fold and assemble is not well understood. Unlike native α7-nAChRs, α7-5HT3 chimeras fold efficiently in HEK cells and do not require chaperones RIC3 or TMEM35A (NACHO) for proper assembly. We investigated the effects of swapping 5HT3 and α7-receptor protein sequences on α7-5HT3R chimera surface expression in mammalian HEK293 or Bosc23 cells, or chimeric receptor function using *Xenopus laevis* oocytes with or without chaperones. α7-5HT3Rs, consisting of human α7-nAChRs with mouse 5HT3 transmembrane domains (TMs) express without chaperones as measured by cell surface alpha-bungarotoxin binding. However, when subunit TMs from α7-nAChRs and 5HT3Rs were mixed, chaperones were required. Substituting the SAP motif prior to the α7-nAChR “Latch” tail sequence for the 5HT3 C-terminal decreased expression relative to α7-nAChRs with chaperones. Chaperone effects on L264 and G265 mutations in M2 were also investigated. Some constructs that express well in HEK293 or Bosc23 cells are nonfunctional in oocytes with or without NACHO. Our data do not support direct binding of RIC3 or NACHO to the α7-nAChR TM4 (M4) region; instead, they emphasize the functional importance of the conserved SAP motif.

## 1. Introduction

How transmembrane proteins fold and assemble is not well understood even though cell surface proteins constitute some 60% of drug targets [1]. Alpha7 nicotinic acetylcholine receptors (α7-nAChRs) are pentameric receptors with each identical subunit having four transmembrane domains (TMs) that fold and assemble in the endoplasmic reticulum (ER). After exiting the ER, α7-nAChRs are processed in other organelles and ultimately transported to the cell surface [2]. Since α7-nAChRs are implicated in several neurological disorders such as Alzheimer’s and Parkinson’s disease how they are expressed becomes important for considering alternative therapies [3,4,5]. Accessory proteins, including chaperones necessary for proper folding and assembly of ion channels, are now viewed as possible targets for therapeutic intervention [6].

α7-nAChRs require specialized chaperones in Human Embryonic Kidney 293 (HEK) and other cells to properly fold and assemble. These include Resistance to Inhibitors of Cholinesterase 3 (RIC3 [7]) or Transmembrane Protein35A (TMEM35A, also known as Nicotinic Cholinergic Receptor Regulator or NACHO [8]). Eiselé et al. [9] demonstrated that a chimeric receptor of the N-terminal of rat α7-nAChR up to TM1 (M1) followed by the rest of mouse serotonin type 3A (5HT3) expresses better than native α7-nAChR in *Xenopus laevis* oocytes. They spliced α7 and 5HT3 at V201 (or V224 if counting the signal sequence, as we will do throughout). Subsequent work showed these α7-nAChR-5HT3 chimeric constructs (5HT3-chimeras) express well in HEK293 cells without chaperones (e.g., [10]), unlike native α7-nAChRs. ^125^I- or fluorescently labeled alpha-bungarotoxin (BGT) binding to surface receptors are excellent but indirect assays for α7-5HT3R and α7-nAChRs expression (e.g., [11]).

In 2002, Halevi et al. [12] showed that RIC3 is a chaperone that increases functional expression of rat α7-n5HT3 M4 sequences (α7-Cα7AChRs in *Xenopus laevis* oocytes as well as Deg-3 and Des-2 worm nicotinic receptors. Gee et al. [13] demonstrated using a variety of rat α7-mouse 5HT3R chimeras that the TMs play a crucial role in receptor expression in HEK tsA201 cells, but RIC3 could not rescue the constructs that did not express on their own. Later, Koperniak et al. [14] found that RIC3 is not present in some cell lines that readily express α7-nAChRs, suggesting the presence of additional specialized chaperones is required for α7-nAChR expression. Kuryatov et al. [15] reached similar conclusions when they found that chemical chaperones were more effective than RIC3 in promoting α7-nAChR expression in HEK cells. Therefore, Gu et al. [8] launched a search for ER-resident proteins as α7 chaperones and found the gene Transmembrane Protein 35A (TMEM35A), which they called Nicotinic AcetylCHOline regulator or NACHO. Curiously, while RIC3 and NACHO appear to function both independently and synergistically in HEK293 cells, knocking out TMEM35A in mice eliminates α7-nAChR expression in the brain (detected by radioactive BGT binding) [16]. In contrast, knocking out RIC3 does not prevent α7-nAChR expression in mouse brain.

It is not well understood how RIC3 and NACHO interact with α7-nAChR [17]. A further limiting factor has been the limited structural information about TM folding in α7-nAChRs. Noviello et al. [18] (as well as Zhao et al. [19]) published an α7-nAChR structure determined by cryo-EM and found an unexpected “latch” helix after α7 M4. In addition, bioinformatic comparisons suggest that an SAP motif (serine, alanine, proline) found just before the α7 latch is highly conserved across species down to the level of *C. elegans*, while the latch itself is not. This suggests a possible functional role for the latch and SAP motifs [17]. Furthermore, Kweon et al. [20] showed that M2 may play a role in NACHO chaperone actions using an α7-5HT3 chimera formed at Threonine 267 (T267).

In the present study, we made chimeras of human α7 and mouse 5HT3A to evaluate these chaperone interactions and their effects on receptor expression and function. Mutations to the α7 SAP motif indicate that substituting P491 plus the latch for the 5HT3 C-terminal in 5HT-chimera allows surface expression in HEK293 or Bosc23 cells without chaperones, but that mutations to the region just before P491 are more problematic. We also made chimeric constructs replacing amino acids 267-318 in human α7 receptor M2 with those of mouse 5HT3 receptor together with mutations in M4.

## 2. Results

### 2.1. Chimera Construction

Sequence homology helped determine the placement of α7/5HT3 splices in chimeras. Bioinformatic comparisons between α7-nAChRs of different species and between human nicotinic receptor subunits demonstrate that α7-nAChRs maintain unique features that are retained throughout evolution in the M4 C-terminal region, including a “SAP” motif before the “latch” helix. Since D468-R469 is conserved in mouse 5HT3A (but R447 is not conserved in human 5HT3A) as well as α7 subunits and all human nicotinic subunits, we chose D446 to make our α7/5HT3 splice location for M4 and also inserted an AgeI site. Other splices were made between the α7 N-terminal and 5HT3 M1–M3 at V226 [5] and at H296 which is common between the end of mouse 5HT3 M3 and the beginning of the human α7 cytoplasmic loop. The final constructs used in this report are listed in Table 1 and Table 2. The reasoning behind the various DNA constructs is explained in Materials and Methods and in Supplemental Figure S1.

### 2.2. α7 M4 and the α7 Tail are Important for NACHO and RIC3 Interactions

Gee et al. [13] observed that α7-5HT3 chimeras containing α7 and 5HT3 TMs failed to achieve functional expression. Even when co-expressed with the chaperone protein RIC3, which typically aids receptor assembly, these hybrid receptors failed to express. The structural or functional incompatibility (i.e., biochemical mismatches preventing proper receptor folding, assembly or stability) caused by mixing TMs from these distinct receptor families remains poorly understood. Specifically, it is unclear which TM regions, residues, or interactions between α7 and 5HT3 domains are responsible for the receptor expression deficit.

**Figure 1:**
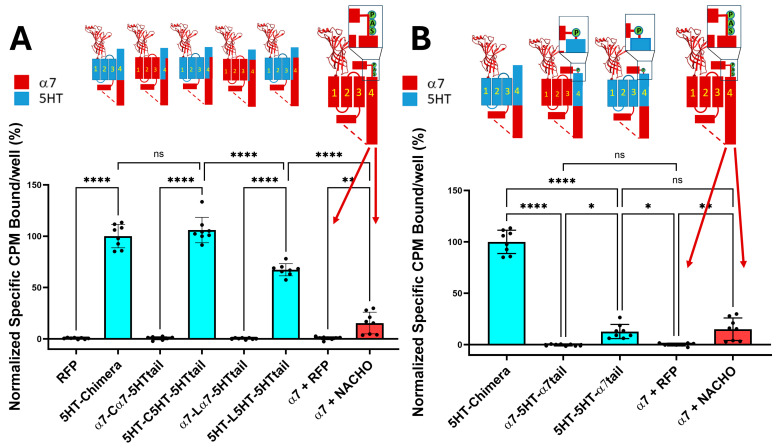
(**A**). Chimeric constructs whose M1–M4 are from 5HT3A expressed without chaperones, unlike those constructs that contain a mixture of α7-nAChR and 5HT3 TM domains. The cartoons show the construct regions in which α7-nAChR (red) is substituted with 5HT3 sequences (cyan). In contrast, α7-nAChR requires NACHO to express on the plasma membrane. The larger α7-nAChR cartoon shows the SAP motif before the latch in light green circles and the dotted lines show the region of the ICD not resolvable by cryo-EM or X-ray crystallography (see, however [21]). The orange bar shows toxin binding after transfection with either RFP or NACHO at a 1:1 ratio with α7-nAChR DNA. Data are from two independent experiments performed in quadruplicate, normalized to the average 5HT-chimera binding in each experiment. (**B**). 5HT3 chimeras with the α7Tail (PNFVEAVSKDFA) express without chaperones if all TMs are 5HT3 in composition. However, 5HT-5HT-α7tail binding in the absence of NACHO is much lower than 5HT-chimera but is comparable with α7-nAChR with NACHO. The data for 5HT-chimera and α7 are identical in both (**A**,**B**). RFP is used as a transfection control. The letters in the cartoons show important amino acids near the latch: S = α7 S489, A = α7 A490, and P = α7 P491. Analyzed by One-way ANOVA in GraphPad Prism 10.5. * *p* ≤ 0.05, ** *p* ≤ 0.01, **** *p* ≤ 0.0001. Data are mean ± SD with *N* = 2 (quadruplicate wells/construct in each of two experiments, data normalized to 5HT-chimera).

As shown in Figure 1A, the 5HT-chimera expressed well without a chaperone protein in HEK293 cells, while the α7-nAChRs require NACHO to express on the plasma membrane. In contrast, constructs containing incompatible TMs, specifically with α7 M1–M3 but facing 5HT3 M4 sequences (α7-Cα7-5HTtail and α7-Lα7-5HTtail), failed to express in HEK293 cells. The same substitutions in chimeras with 5HT3 M1–M3 did express on the plasma membrane. These data support the hypothesis that substituting 5HT3 M4 amino acids that project into the lipid bilayer with α7 residues will not prevent chimeric expression. Figure 1B explores the effect of adding an α7-tail (PNFVEAVSKDFA) onto 5HT3 M4 with 5HT3 M1–M3. The 5HT-5HT-α7tail receptor expresses about the same in HEK293 cells as α7 with human NACHO in 1:1 ratio. However, α7-5HT-α7tail does not express in the absence of chaperones.

### 2.3. Quantification of Radioactive Binding Assay vs. Fluorescent Toxin Binding

We aimed to compare the gold standard of radioactive toxin binding to that of fluorescent toxin binding. Isotope dilutions with freshly made ^125^I-BGT and varying concentrations of unlabeled toxin were determined by constructing a plot of 1/CPM bound vs. concentration of competing unlabeled BGT (Supplementary Figure S2A). For comparison, a series of competition assays with varying concentrations of unlabeled toxin were performed in a fluorescent binding assay in quadruplicate and were further analyzed using ImageJ. These showed linear competitions between Alexa Fluor™ 647 conjugated BGT (F-BGT) and unlabeled BGT (Supplementary Figure S2B). The same concentrations of unlabeled toxin that displaced radiolabeled toxin displaced the fluorescent toxin. Representative 4× and 20× images are shown in Supplementary Figure S2C. We also investigated how often transfecting with three plasmids allowed expression in the same cell by using three fluorescent constructs simultaneously (5HT-chimera bound with F-BTX, RFP, and GFP; Supplementary Figure S3) and found values ranging from 13 to 91%.

### 2.4. Mixing M4 with Different TMs M1-M3 Does Not Allow for Plasma Membrane Expression, Even with Chaperones

Figure 1 shows that for the chimera receptors to express on the plasma membrane of HEK293 cells in absence of chaperone proteins, M1–M3 must be derived from the same receptor (α7-nAChR or 5HT3A) as M4, that and the α7 tail decreases chimera expression. We repeated these studies using F-BGT binding to Bosc23 cells transfected with the same constructs as in Figure 1 but under four conditions; various constructs were transfected with a 1:1 ratio with GFP (control), with NACHO and GFP 1:0.75:0.25, with GFP and RIC3 1:0.75:0.25, or with NACHO and RIC3 1:0.75:0.25. We previously reported that a 3:1 ratio between NACHO and RIC3 gave the highest synergy, increasing α7-nAChR expression in HEK293 cells [16], so we kept those ratios to test for synergistic effects.

**Figure 2:**
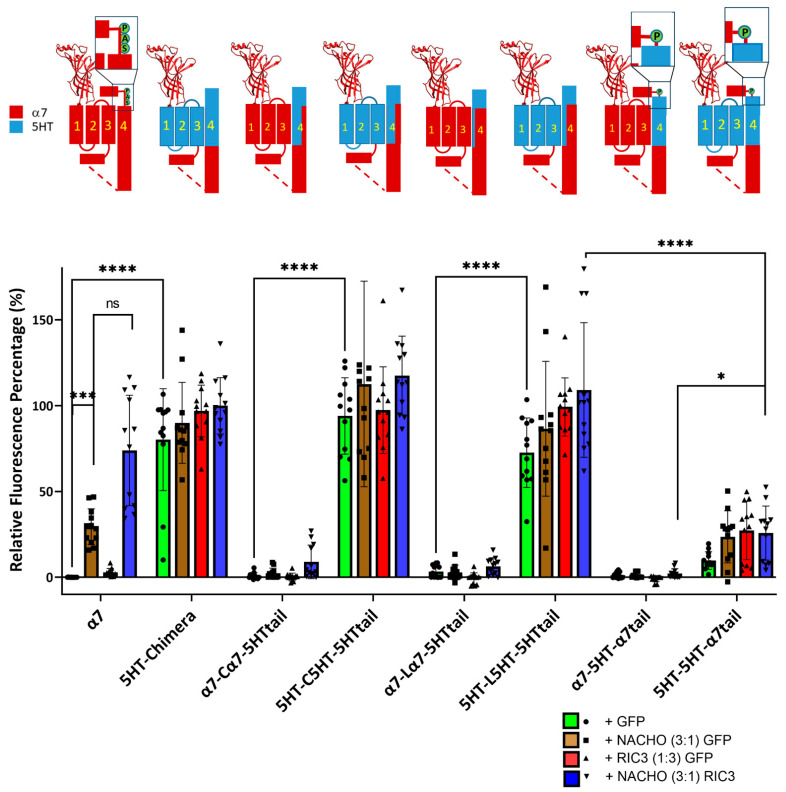
Incompatibility between M1-M3 and M4 reduces NACHO function and synergistic effects of NACHO with RIC3. Relative fluorescent intensity normalized per well using α7 + GFP (negative control) and 5HT-chimera + GFP (positive control). The cartoons are as in Figure 1. * *p* < 0.05, *** *p* < 0.001, and **** *p* < 0.0001, ns = not significant. Brown–Forsythe and Welch One-Way ANOVA. Data are mean ± SD (*N* = 3 experiments, quadruplicate samples each).

Consistent with Figure 1, constructs with a mixture of α7-nAChR and 5HT3 TM domains failed to show significant surface expression in the absence of chaperones (e.g., α7-Cα7-5HTtail + GFP). The α7-5HT-α7tail also does not express in the absence or presence of chaperone proteins (Figure 2). This suggests an important role of M4 in receptor expression: TM 1–3 need to match the region of M4 projecting towards those TMs for plasma membrane expression in the absence of chaperones.

In contrast, constructs in which M1–M3 are compatible with M4 (i.e., from the 5HT3 receptor), expression was high in the absence of chaperones and was largely unaffected by chaperones. Taken together, these data suggest compatibility between M1–M3 and M4 is critical for α7 chaperone-mediated surface expression, and the fluorescence results mirror the data seen using radioactive toxin binding in Figure 1.

### 2.5. Construct Expression in Oocytes

**Figure 3:**
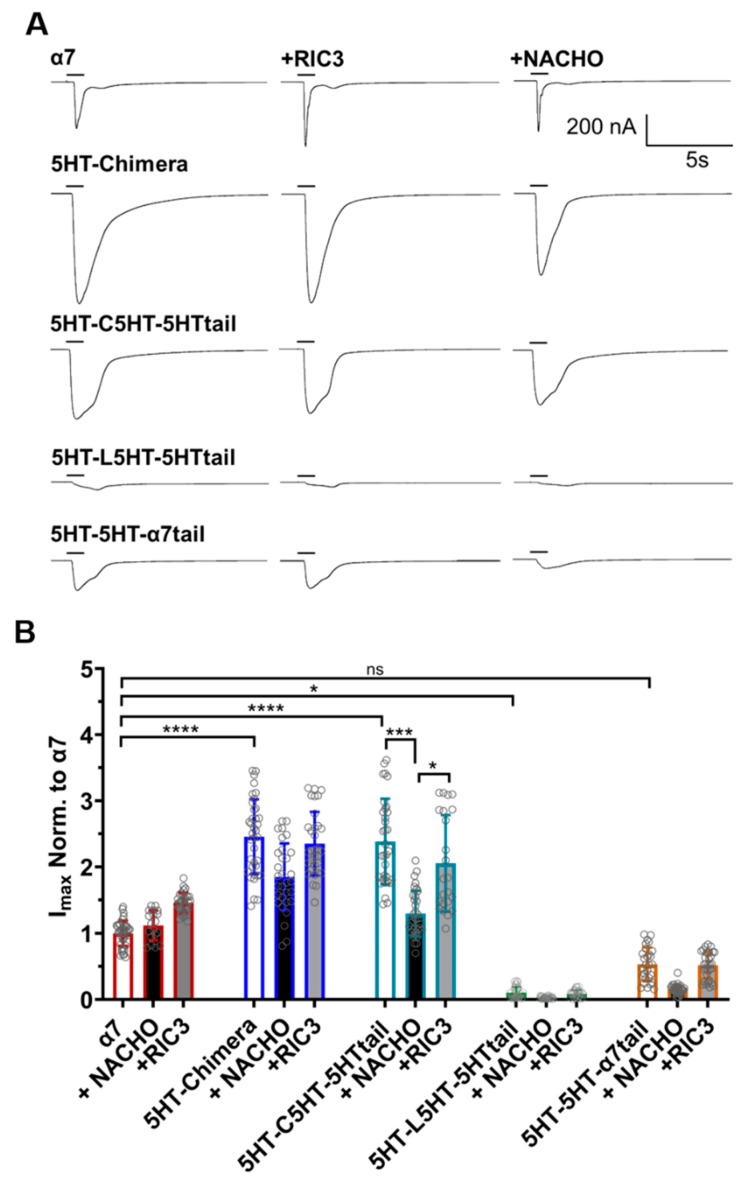
RIC3 or NACHO have minimal effects on acetylcholine inducted function on α7-nAChR and 5HT3 chimeric constructs. Each receptor was individually expressed in oocytes and recorded using TEVC six days post cRNA injections. Data were normalized to the α7-nAChR control group. (**A**): Representative traces of the responses showing the faster desensitization and/or closing of wild-type α7-nAChRs compared to the chimeric constructs following 1s acetylcholine (10 mM) administration. (**B**): RIC3 slightly increases peak currents for α7-nAChR in oocytes but has no significant effects on any other construct. NACHO has no effect on α7-nAChR peak currents but significantly decreases the peak currents of the 5HT-C5HT-5HTtail chimera. Gray circles represent normalized peak currents in individual oocytes compared to 10 mM acetylcholine in the α7-nAChR control group. * *p* < 0.05, *** *p* < 0.001, and **** *p* < 0.0001, ns = not significant. Two-way ANOVA with Tukey’s multiple comparisons test. Data are mean ± SD (*N* = 3–4, number of oocytes per group *n* = 19–39).

A different picture emerges for these constructs when measuring functional expression in oocytes (Figure 3). α7-nAChR expresses well in oocytes in the absence of RIC3 or NACHO addition. The 5HT-chimera and 5HT-C5HT-5HT tail currents were significantly greater than α7-nAChRs. Surprisingly, 5HT-L5HT-5HTtail and 5HT-5H-α7tail showed significantly less or similar levels of function, respectively, compared to α7.

In HEK293 cells, 5HT-L5HT-5HTtail is expressed as well as the 5HT-chimera (Figure 2), suggesting that if this construct expresses similarly in oocytes, then it is functionally impaired. RIC3 or NACHO could not further improve expression significantly for any of the chimeras, except for 5HT-C5HT-5HTtail, as there was no effect on acetylcholine peak currents. The 5HT-C5HT-5HTtail, when expressed with NACHO, reduced the peak response to levels similar to α7-nAChR, and was the only construct to show sensitivity to NACHO. It seems likely that oocytes have their own endogenous chaperone proteins, as α7-nAChRs readily express without exogenous chaperone additions.

### 2.6. The SAP Motif Decreases Expression of 5HT3 TM Constructs

**Figure 4:**
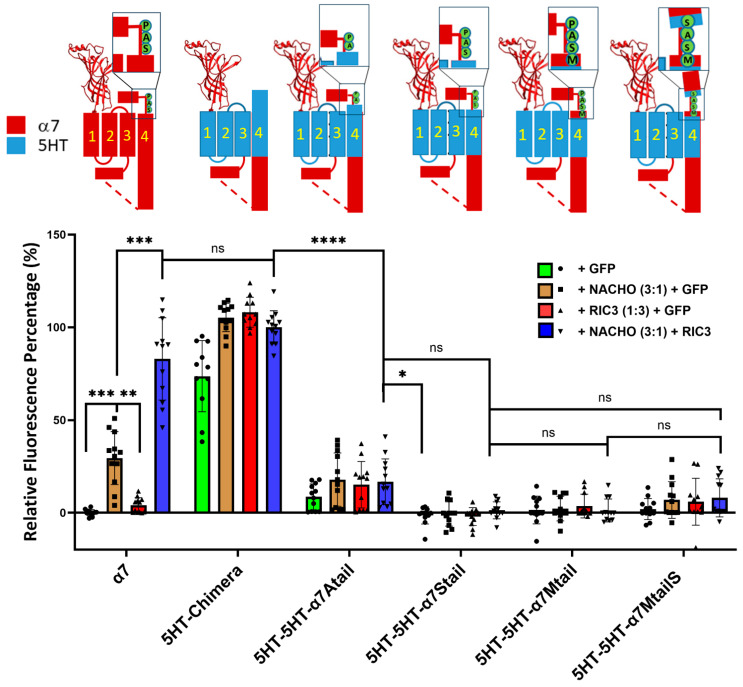
Replacing residues in the 5HT-5HT-α7tail construct (see Table 1) prior to the proline in the SAP motif, or replacing P491 with serine, largely blocks expression and chaperone actions. Relative fluorescent intensity was normalized using α7 + GFP (negative control) and α7-5HT3 chimera + NACHO + RIC3 (positive control). The letters in the cartoons show the differences in sequence: M = α7 M488, S = α7 S489, A = α7 A490, P = α7 P491, and in the α7MtailS cartoon, the S on the cyan background shows the P491S mutation. Data are mean ± SD (*N* = 3 experiments each performed in quadruplicate). One-way ANOVA * *p* < 0.05, ** *p* < 0.01, *** *p* < 0.001, and **** *p* < 0.0001, ns = not significant.

To further investigate the effects of the SAP motif in chaperone actions, we mutated the 5HT3-5HT-α7tail chimera starting from V487, T488, L489, and W490 (Table 1) by substituting the SAP residues one at a time starting with W490A (Table 1). We found that surface expressions of those chimeras were decreased significantly, and neither NACHO, RIC3, nor the combination of both could rescue surface expression (Figure 4), although there was slight expression of 5HT-5HT-α7Atail. ColabFold-AF2 predictions suggest that the major difference in structure between 5HT3-5HT-α7tail vs. 5HT3-5HT-α7Mtail is the orientation of the proline prior to the latch (Supplementary Figure S4). The bulky W490 in 5HT3-5HT-α7tail is predicted to project into the opening between M1 and M3, but why these structural changes would affect receptor expression is not immediately clear. Similarly, there seems to be relatively minor structural differences between the 5HT-chimera and 5HT3-5HT-α7MtailS (Supplementary Figure S5) besides the differences in the C-terminal tails, but in this case, W490 is predicted to project directly towards M3. Finally, we also made similar constructs that instead have α7 M1-M3 and 5HT3 M4 with an α7 latch (e.g., α7-5HT-α7Mtail in Table 1), but these expressed poorly with or without chaperones (Supplementary Figure S6). Collectively, these data emphasize the importance of the tail in chaperone-induced α7-nAChR plasma membrane expression.

### 2.7. RIC3 Synergy is Enhanced with NACHO Interaction Within the α7 M1 and M2 Domains

**Figure 5:**
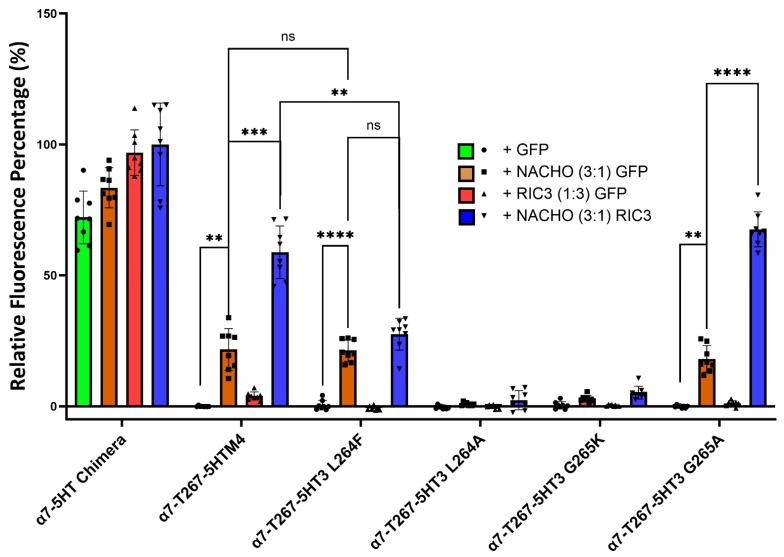
Mutations to L264 and G265 give different results depending on the substitution. Construct model sequences are listed in Table 2. One-way Brown–Forsythe and Welch ANOVA. Data are mean ± SD (N = 2 experiments, quadruplicate samples each) normalized to α7-5HT chimera +NACHO + RIC3 as 100% and α7-T267-5HTM4+GFP as 0%. ** *p* < 0.01, *** *p* < 0.001, and **** *p* < 0.0001, ns = not significant.

Kweon et al. reported two amino acids (L264 and G265) in M2 that critically determine whether NACHO aids α7-nAChR expression [20]. Their T267-5HT3 chimera has an α7 N-terminal and α7 M1, a partial M2 split between α7 and 5HT3 at T267, and the rest is all 5HT3. To further investigate the function of L264 and G265 in a slightly different configuration (our ICD is α7, not 5HT3), we substituted amino acids 1-267 in the 5HT-chimera to α7 residues to create the α7-T267-5HTM4 chimera. Next, we mutated amino acids L264 and G265 in the α7-T267-5HTM4 chimera to the corresponding amino acids (F264 and K265) found in the 5HT3 receptor (Table 2, Figure 5 and Figure S7) and co-transfected them with NACHO and/or RIC3. Like α7-nAChR, α7-T267-5HTM4 chimera significantly expresses in the presence of NACHO, and RIC3 synergistically enhances that expression. However, unlike what Kweon et al. reported [20], NACHO alone caused no difference in α7-T267-5HT3 L264F expression from that of α7-T267-5HTM4, but the synergistic effects of RIC3 were lost. In contrast, α7-T267-5HT3 G265K expression with both NACHO and the NACHO/RIC3 combination was significantly decreased as reported by Kweon et al. [20].

F264 and K265 are very bulky residues compared to L264 and G265, and therefore we replaced these with alanine, which has a smaller side chain. The L264A mutation completely lost surface expression with or without chaperones. In other words, L264 does not tolerate alanine substitution, but L264 tolerates substitution with phenylalanine at the cost of losing RIC3’s ability to potentiate the effects of NACHO. In contrast, expression of the G265A mutation is indistinguishable from the α7-T267-5HTM4 parent construct in the presence of NACHO, and like both α7-T267-5HTM4 and α7-nAChR, RIC3 synergistically potentiates the effects of NACHO.

**Figure 6:**
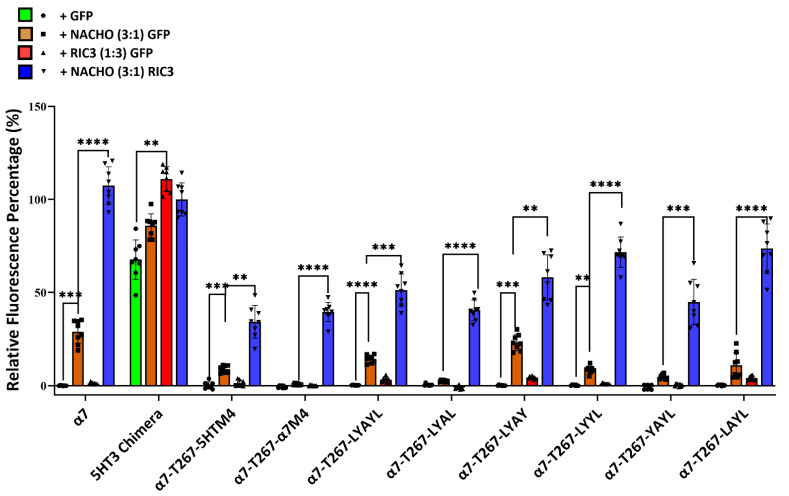
Inability to determine the cause for 5HT3-α7 TM incompatibility using T267 chimeras. Relative fluorescent intensity normalized for each well using α7-nAChR + GFP (negative control) and 5HT3 chimera +NACHO + RIC3 (positive control). One-way ANOVA. Data are mean ± SD (*N* = 2 experiments, quadruplicate samples each). Comparisons are to show the effects of the chaperones on individual mutated receptors. ** *p* < 0.01, *** *p* < 0.001, and **** *p* < 0.0001.

Figure 1 and Figure 2 suggest that M4 must match M1-M4 to allow significant expression with or without chaperones. But Kweon et al.’s [20] α7-T267-chimera construct and our similar α7-T267-5HTM4 construct (Figure 5) show that this is not the case, and suggest that M4 only needs to match M3. To test this, we generated α7-T267-5HTM4 (M3 and M4 are both 5HT3 TMDs) and α7-T267-α7M4 (M3 and M4 differ). Suspecting that α7-T267-α7M4 would not express, we also generated an LYAYL series (Supplementary Figure S1C) that replaced amino acids in a7M4 that project towards M3 with those from 5HT3M4. However, unexpectedly α7-T267-α7M4 expressed as well as α7-T267-5HTM4 in the presence of NACHO and RIC3 (Figure 6), suggesting there is no clash between M3 and M4 in T267 constructs. This made it impossible to find single amino acids responsible for the incompatibility between M3 and M4 observed when M1-M3 were all either 5HT3 or α7-nAChR. Moreover, the synergistic effects of NACHO and RIC3 were preserved in all α7-T267 constructs tested in Figure 6.

## 3. Discussion

α7-nAChRs are implicated in several neurological disorders such as schizophrenia, spectrum disorders, and both Alzheimer’s and Parkinson’s disease [3,4,5], and are considered appropriate targets for drug therapy [22]. However, ligands binding directly to α7-nAChRs have not been useful in clinical applications so far. Some investigators are now looking at accessory proteins such as RIC3 and NACHO that affect α7 nAChR assembly and surface expression as alternative therapies [6,23]. For example, evidence for an α7 role in Alzheimer’s is very strong [24,25]. Amyloid beta (Aβ), an Alzheimer’s disease plaque protein, binds to α7-nAChRs with low pM affinity [26] and both are found together in the plaques characteristic of the disease. The reported physiological effects of Aβ binding to α7-nAChRs are varied (reviewed by [24]), but the ability of α7-nAChRs to regulate intracellular calcium [27] raises a variety of possible etiologies, including excitotoxicity. Chaperones such as NACHO may play a direct role in Alzheimer’s by interacting with another protein, Ly6H, that antagonizes the effects of the chaperone on α7-nAChR expression [28].

Despite the interest in the role of α7-nAChRs in neurological diseases, relatively little is known about how chaperones interact with the receptor during folding and assembly in the endoplasmic reticulum (ER). Evidence shows that two of these chaperones, RIC3 and NACHO, aid α7-nAChR assembly by independent mechanisms [6,8,16,20]. Due to advances in α7-nAChR structural analysis [18,19], we now know much more about what the final assembled α7 pentamer looks like, but the many steps required for assembly and exactly how these chaperones influence this process remain obscure. Some broad outlines are known for nicotinic receptors. Receptor assembly and trafficking are inefficient processes with synthesis, folding and initial pentameric assembly taking place in the ER, with additional modifications in the Golgi before export via vesicles to the plasma membrane (e.g., [2]).

A central mystery about RIC3 is how a family of intrinsically disordered proteins with little sequence homology across species (except for some homology in transmembrane domains and coiled-coil domains) [29,30] functions to assist folding and assembly of α7-nAChR subunits from a variety of species [17]. Wang et al. [31] hypothesize that RIC3 pulls individual pre-folded α7-nAChR subunits together through dimerization of RIC3 coiled-coiled domains. How NACHO acts to assemble receptors is also not clear. Kweon et al. [20] hypothesize that NACHO does not directly contact α7-nAChR subunits, but instead pulls together other chaperones such as oligosaccharide transferase and calnexin to interact as α7-nAChR subunits enter the ER. This results in accelerated glycosylation of α7-nAChR subunits and greater assembly. A recent study suggesting a role for NACHO in α7 receptor glycosylation found that Chinese Hamster Ovary (CHO) cells lack NACHO, but CHO cells can assemble α7-nAChR subunits with the help of RIC3. However, these receptors, although functional, showed deficiencies in glycosylation and receptor trafficking [32]. On the other hand, a recent preprint by Hooda et al. [33] finds Cryo-EM evidence that NACHO participates in the folding and assembly of GABAa α1 subunits by direct binding to the GABA α1 TMs M2 and M3. Further, when they altered contact points between NACHO and the GABA subunit, the mutated NACHO did not properly assemble α7-nAChR subunits in HEK cells, suggesting that NACHO also directly contacts α7 TMs during assembly.

An advantage of α7-nAChRs for studying multimeric assembly is the similarities with 5HT3 receptors, which express well as homo-oligomers in most cells without specialized chaperones and are very similar in structure. It has been known since 1993 that α7-5HT3 chimeras express better than α7-nAChRs in a variety of cell systems [9]. Gee et al. [13] were among the first to use α7-5HT3 chimeras as a tool to identify regions within the α7-nAChR subunit where RIC3 exerts its chaperone effects. However, they discovered that combining TMs from α7 and 5HT3A receptors disrupts chimeric receptor expression, and that RIC3 is unable to improve the expression deficit. Kweon et al. [20] also used α7-5HT3 chimeras and localized NACHO effects to eight amino acids localized to the base of α7 M2. Our results with the T267-series largely corroborate those results, albeit with an α7 ICD compared to the 5HT3 ICD of Kweon et al. However, our results do not support the proposal of Kweon et al. that L264 and G265 are sites of NACHO interaction with α7 subunits (Figure 5). In our constructs that differ primarily from those of Kweon et al. by having an α7 ICD instead of 5HT3, the L264F mutation had no effect on NACHO but lost the synergism with RIC3 and the G265A mutation was indistinguishable from α7-T267-5HTM4 with full RIC3 synergism with NACHO. Clashes by a bulky side chain in the G265K mutation may explain the assembly block. Also, superimposing an α7 subunit over a GABAa α1 subunit from the model by Hooda et al. [33] strongly suggests that L264 and G265 are on the opposite side of M2 from where it makes NACHO contact (Supplementary Figure S8).

Returning to the question of incompatibility when mixing M1–M3 and M4 from α7 and 5HT3 receptors, we find results similar to Gee et al. [13] that mixing the TMs results in constructs that do not express without chaperones and that RIC3 is not able to rescue expression (Figure 1 and Figure 2). However, NACHO rescued construct expression, and RIC3 showed synergism with NACHO (Figure 2). Since the highest sequence conservation is in the RIC3 transmembrane region across species, and α7 M4 is the most exposed transmembrane region in the assembled receptor, we also tested the effects of swapping α7 amino acids for those 5HT3 residues that project into the lipid bilayer in M4 (C and L constructs in Figure 1 and Figure 2 and Table 1). RIC3 synergism with NACHO was largely blocked in these constructs (Figure 2). Conversely, swapping 5HT3 residues for α7 amino acids in M4 when M1-M3 were 5HT3 did not produce constructs that required RIC3 for surface expression.

Since α7-T267-5HTM4 expresses well with NACHO and has an α7M1, a mixed M2, and 5HT3 M3 and M4, we thought we could explain why the combination of 5HT3-M4 with α7-M3 does not express without chaperones because of a clash between M4 and M3 sidechains. Mesoy et al. [34] performed alanine substitution analysis in 5HT3A M4 and found five amino acid mutations that substantially blocked 5HT3A receptor function measured by a voltage-sensitive dye in HEK293 cells: D461A, Y468A, Y475A, W483A, and W486A (5HT3 numbering with signal sequence). These correspond to D468, Y475, Y482, W490, and W490 in α7-5HT3 chimeras. Three (Y475, Y482, W490) of the seven amino acids we identified as potential incompatibility sites between 5HT3 M4 and α7 M3 (Supplementary Figure S1C) block wild-type 5HT3 function if substituted with alanine. However, D468 is common to both wild-type 5HT3AR (D434) and 5HT-chimera, so we had no direct data on this substitution. Mesoy et al. [34] find that the similar mutant 5HT3AR D434N can be rescued with RIC3, although D434A cannot. Noviello et al. [18] report that α7 D468 makes a required salt bridge with K261 in M2, but this is not conserved in the 5HT-chimera. Similarly, da Costa Couto et al. find D468 is required for α7-nAChR assembly when assayed by alanine substitution analysis [35]. However, our hypothesis also predicted that α7-T267-α7M4 should not express due to clashes, but that is not what happened (Figure 6). Therefore, our current working hypothesis is that M1 and/or the lower half of M2 specifies whether chaperones are needed to express. Thus, if M1 and ½ M2 are 5HT3 sequences, no chaperone is needed for receptor expression, but if they are α7, NACHO is needed, and RIC3 should synergize. However, why this would be so is not clear. Furthermore, this interpretation is completely compatible with Kweon et al.’s [20] result that the NACHO determinant lies between G265 and T267 in α7-nAChR.

As noted in a recent review [17], the SAP motif before the latch helix is evolutionarily ancient in α7-nAChRs. Noviello et al. [18] report that the “latch turn” consisting of the “AP” (A490-P491) part of the SAP motif is essential for channel gating and interactions with the Cys-loop. They found that a P491A mutation or deletion of A490 in α7-nAChR leads to receptors that express on cell surfaces but are nonfunctional. However, we find that W490 in 5HT3-5HT-α7tail is a functional replacement for A490 in both cell surface expression (Figure 1 and Figure 2) and in functional expression in oocytes (Figure 3) if the rest of M4 has a 5HT3 sequence. Attempts to replace more of 5HT3 M4 with additional amino acids from the SAP sequence led to constructs that failed to show surface expression or expressed poorly in Bosc23 cells (Figure 4). For that reason, electrophysiological testing in oocytes was not pursued further.

To our knowledge, our recordings of human α7 with NACHO represent the first reported recordings of this chaperone’s effects on vertebrate α7-nAChR and α7-5HT3 chimeras in oocytes. Unexpectedly, NACHO in oocytes either had little effect (α7-nAChR) 620 or tended to suppress function (α7-5HT3 chimeras), quite different from surface expression in HEK293 cells. However, this is not the first instance that a chaperone has different effects in a cell line versus oocytes. For instance, RIC3 is reported to markedly suppress α3β4 and α4β2 receptor function in oocytes [29] but strongly enhances expression in HEK tsA201 cells [36]. This may be due to a different mix of endogenous chaperones in oocytes compared to HEK cells. We did not observe a marked enhancement of α7-nAChR function induced by RIC3. Unexpectedly, 5HT-L5HT-5HTtail chimera had little function in oocytes, but good surface expression in HEK293 cells. Further experiments are necessary to determine if this is due to a lack of expression in oocytes or a lack of function in HEK293 cells. Interestingly, a recent report suggests that *Drosophila* NACHO strongly supports *Drosophila* Dα1/Dβ1 nicotinic acetylcholine receptors in oocytes [37], but these receptors are different from the *Drosophila* α7 analog ACR-16. Also, NACHO allows oocyte expression of *Apis mellifera* α6 nAChRs, although this insect subunit has a RAP motif instead of the α7 SAP motif [38]. Finally, addition of NACHO and RIC3 allows functional expression of otherwise non-functional mutant rat α4β2 receptors substituted with alanine in selected M4 residues [39]. Together, these data suggest that RIC3 and NACHO interact with C-terminal regions of various types of receptors during folding and assembly.

We have some caveats about our results and within the field of chaperone effects in general. We would like to believe that the ecosystem of ER chaperones within a specified cell line is constant so that results from one lab are readily comparable with another. However, Hooda et al. [33] report some strains of their HEK cells express a measurable basal level of NACHO, something we have never seen (e.g., Supplementary Figure 1 in reference [16]). Additionally, a cell line should be unchanging. We previously reported that RIC3 promotes significant α7-nAChR expression in HEK cells without NACHO (e.g., Figure 3 in reference [16], similar to effects reported for mouse RIC3 by Wang et al. [31], but in this study we see only synergistic effects of RIC3 when expressed with NACHO, like that observed by Kweon et al. [20]. This is one reason we switched to Bosc23 cells since results by Wang et al. [31] suggest that those cells might be more conducive to studying RIC3 effects on α7-nAChRs. However, besides a slightly greater ease of transfection, we see little difference between Bosc23 and HEK cells, although small differences in innate chaperone actions may be difficult to detect between different cell lines or under slightly different culture conditions.

Additional caveats include the variability of co-transfection of receptor and chaperone plasmids between experiments. Since RIC3 reliably enhances the effects of NACHO on α7-nAChR assembly, this strongly suggests that sufficient DNA is being transfected but our attempts to estimate the number of cells that can be triple co-transfected with fluorescent proteins (Supplementary Figure S3) suggest variability between experiments, and the observed effects of the chaperones could be underestimates. We make no claims about the validity of the structures predicted by AlphaFold2 but include them in the supplemental data to show that small changes in the predicted structures of receptor C-terminal regions can make big differences in cell surface expression. Also, functional expression in oocytes is not equivalent to surface expression in HEK/Bosc23 cells detected by toxin binding. Finally, the toxin binding assay is an indirect measure of receptor assembly since assembly takes place in the ER, and many additional steps are involved in cell surface expression. We are investigating alternative methods for measuring receptor folding, assembly, and degradation but these are not ready to report here.

## 4. Materials and Methods

### 4.1. Bioinformatic Comparison and Molecular Modeling

Bioinformatics studies were performed using NCBI BLAST versions 2.11.0-2.16.0 [40]. Molecular modeling can be divided into two epochs, which were performed prior to the α7-nAChR latch helix structure determination [18], and those performed afterward. Models based on NMR data and homology with 5HT3 receptors were developed using Modeller [41] and Matchmaker [42] within UCSF Chimera 1.17 and 1.18 [43], and later, ChimeraX 1.7–1.10 [44]. Models were rendered using ChimeraX [44] and structural predictions were performed by ColabFold-AF2 [45,46] and AlphaFold2 [47], accessed within ChimeraX 1.8–1.10.

### 4.2. DNA Constructs and Plasmids

Roger Papke and Jon Lindstrom generously provided the human α7 subunit DNA in pCI-neo. Genscript (Piscataway, NJ, USA) synthesized a human α7-5HT3 chimera (5HT-chimera in Table 1) consisting of N-terminal α7, mouse 5HT3 transmembrane domains 1–3 (M1–M3), an α7 cytoplasmic loop, followed by the mouse 5HT3 M4 and C-terminal extracellular tail using sequences from NM_000746.5 (human Chrna7) and NM_013561.2 (mouse 5HT3A). The splice sites were V224 (human equivalent to Eiselé et al. [9], numbering includes the signal sequence) between the α7 N-terminal and mouse 5HT3 M1–3, H319 between the end of mouse 5HT3 M3 and the human α7 cytoplasmic loop, and D468 at the end of the human α7 cytoplasmic loop and the beginning of mouse 5HT3 M4.

Genscript inserted the synthetic 5HT-chimera gene between NheI and NotI in pCI-neo with additional MluI and XhoI sites inserted before NotI. Using A Plasmid Editor (APE, versions 2.0.46-3.1.7), we designed silent restriction sites in the synthetic gene at V224 (BclI, [9]), R227 (SalI), S258 (AccIII), V295 (Bstz171), I314 (BsiWI), and D468 (AgeI).

We generated two chimeras with mixed human α7 and mouse 5HT3A transmembrane domains as follows: hα7-α7M1-3-α7loop-5HT3M4 was generated by PCR using IDT (Coralville, IA, USA) forward primer GTTGGTGAATTCTTCTGGGCATTGC and reverse primer ACAGCAACCGGTCCACCACACAGGCGGC on a human α7 template and inserting the product between EcoRI and AgeI sites in 5HT-chimera. Similarly, we inserted human α7 PCR product created using IDT forward primer TGGTGGACCGGTTGTGCCTCATGGCCTTCTCG and reverse primer CTCGAGACGCGTTTACGCAAAG-TCTTTGGACACGG between AgeI and MluI sites in hα7-m5HT3M1-3-hα7loop-5HT3M4 chimera to create hα7-m5HT3M1-3-hα7loop-α7M4. These two constructs were starting materials for further modifications. Sanger sequencing verified all DNA constructs (Genewiz, South Plainfield, NJ, USA). Genewiz made further constructs by splicing different sequences between AgeI and MluI sites in hα7-α7M1-3-m5HT3M4 or hα7-m5HT3M1-3-hα7loop and M4 constructs as indicated in Table 1. Genewiz generated α7-T267-5HTM4 (Table 2) from the 5HT-chimera by cutting with EcoRI at V132 and AgeI at D468 and inserting hα7 sequence up to T267, then 5HT3 sequence up to H318 and then reverting to α7 sequence until the AgeI site at D468. Silent restriction sites were inserted at R227 (AccIII), S251 (BsiWI), P348 (XmaI), F389 (SacII), and I436 (XbaI). Genewiz generated additional T267 constructs (Table 2) by inserting M4 sequences after cutting at AgeI (D468) and at an MluI site inserted after the stop codon. Human RIC3 isoform 1 in pcDNA3.1 was purchased from Genescript and subcloned into prep9KB plasmid as previously described [16]. Human TMEM35A in pCMV6 was purchased from Origene as previously described [16].

Prior to publication by Noviello et al. [18], structural information of the TM domains for intact α7-nAChR was not available. We hypothesized that clashes between amino acid side chains are the cause of incompatibility between α7 and 5HT3 TMs. Therefore, substituting amino acid side chains that project into lipid membranes should not make any difference for assembly but might alter chaperone recognition. We used molecular modeling to find the amino acid side chains in 5HT3 M4 that project away from M1 and M3. Those residues projecting directly away from M2 into the lipid bilayer were considered sites for conservative swaps (“C”) between α7 and 5HT3 amino acids, while those that project into the membrane more obliquely were considered less conservative (“L”) (Supplementary Figure S1A,S1B). The then available data on 5HT3 TMs and NMR data (PDB 2MAW) on simulated α7 TMs correlated well, and the models overlapped including PDBs 7KOO and 7EKI, when those became available [18,19]. The T267 constructs in Table 2 are based on the results of Kweon et al. [20], except that our constructs have α7 sequences in the ICD.

### 4.3. Cell Lines

Bosc23 and HEK293 cells from ATCC were grown in Dulbecco’s Modified Eagle Medium (Gibco high glucose DMEM with pyruvate, Thermo Fisher, Waltham MA, USA) supplemented with 10% fetal bovine serum (Gibco heat inactivated, Thermo Fisher, Waltham MA, USA) and 1% 100 u/mL penicillin/100 ug/mL streptomycin (MP Biomedicals, Irvine CA, USA) and maintained at 37 °C in 5% CO_2_. Cells were then passaged at a 1:10 dilution and used at low passage numbers.

### 4.4. Reagents for Two-Electrode Voltage Clamp Electrophysiology Oocyte Recordings

Acetylcholine (ACh), atropine, and other reagents used to make buffers were purchased from Sigma-Aldrich (St. Louis, MO, USA), unless otherwise specified. Fresh solution stocks were made daily and diluted as required. To prepare for cRNA synthesis, α7, α7-chimera, and α7-5HT3A mutant chimera construct cDNAs were linearized using the restriction enzyme (New England Biolabs, Ipswich, MA, USA) MfeI. Chaperone proteins RIC3 and NACHO cDNAs were linearized using restriction enzymes NarI or BclI, respectively. Following cDNA linearization, samples were treated with proteinase K (30 min at 50 °C) (New England Biolabs) and purified using Qiagen’s (Germantown, MD, USA) PCR clean-up kit. cRNAs were transcribed using the T7 mMESSAGE mMACHINETM High Yield Capped RNA Transcription Kit (Thermo Fisher Scientific, Waltham, MA, USA). The cRNA purity was confirmed by measuring the 260/280 ratio via a Nanodrop and by visual inspection of samples run on a 1% agarose gel stained with ethidium bromide. The samples were sub-aliquoted and stored at −80 °C.

### 4.5. Oocyte Preparation and cRNA Injection

All efforts were made to reduce animal discomfort and the number of animals used for our experiments. *Xenopus laevis* oocytes were purchased from EcoCyte Bioscience (Austin, TX, USA). Oocytes were stored in sterile incubation buffer (82.5 mM NaCl, 2.5 mM KCl, 1 mM MgCl_2_∙2H_2_O, 1 mM Na_2_HPO_4_, 5 mM HEPES, 600 µM theophylline, 2.5 mM Na pyruvate, 50 U/mL penicillin, 50 µg/mL streptomycin, 50 µg/mL neomycin, 50 µg/mL gentamycin sulfate, pH to 7.5 using NaOH) in a 13 °C incubator.

Using only stage V oocytes, α7-nAChRs, or 5HT3A chimera constructs in the absence and presence of RIC3 or NACHO were expressed in *Xenopus laevis* oocytes using cRNA microinjections. A total of 40 ng of cRNA for the α7-nAChR or 5HT3 chimeric constructs resulted in excellent expression of these receptors. To study the chaperone proteins, 40 ng of either RIC3 or NACHO cRNA were co-injected with each α7-5HT3A chimera construct or α7-nAChR. In all cases, 81 nl of cRNA were injected into each oocyte by a pulled micropipette with an outer diameter of about 40 µm. Injected oocytes were incubated for six days prior to recording.

### 4.6. Two-Electrode Voltage Clamp Electrophysiology

We used two-electrode voltage clamp (TEVC) and *Xenopus laevis* oocytes expressing nAChR or 5HT3A chimera constructs in the absence or presence of RIC3 or NACHO to gauge changes in macroscopic function. Experiments were performed similarly to those reported previously [48]. Briefly, to evaluate receptor function, each oocyte was voltage clamped at −70 mV with an Axoclamp 900A amplifier (Molecular Devices, LLC, Sunnyvale, CA, USA). Initially, to determine the ACh concentration that induced the maximal response (I_max_), ACh concentration-response profiles were recorded (1 µM–10 mM) from each receptor group as controls. Application of 10 mM ACh induced the maximal response in all groups. To measure potential changes in I_max_, 10 mM ACh was applied for 1s followed by 26s of OR2 buffer wash (92.5 mM NaCl, 2.5 mM KCl, 1 mM MgCl_2_∙5H_2_O, 1 mM CaCl_2_∙2H_2_O, 5mM HEPES, pH to 7.5 using NaOH). Atropine sulfate (1.5 µM) was added to all recording solutions to block potential muscarinic responses. ACh and buffer were applied to clamped oocytes using a 16-channel, gravity-fed, perfusion system with automated valve control (AutoMate Scientific, Inc., Berkeley, CA, USA). Five to twelve oocytes were recorded for each receptor group and experiment.

The recording apparatus used minimized post-valve tubing length and a custom manifold to reduce dead volume. Data acquisition and analysis were performed using pClamp 10.7 software (Molecular Devices, LLC, San Jose, CA, USA). TEVC recordings were sampled using a 10 kHz low-pass Bessel filter and a 40 Hz high-pass filter to suppress DC offset. Thin-wall capillary glass (World Precision Inst, Sarasota, FL, USA) was used to pull recording electrodes. Electrodes were filled with 3 M KCl and had a final electrode of 0.5–10 MΩ. Oocytes with leak currents that were larger than −100 nA were discarded. Peak current amplitude (I_max_) values were determined from individual oocytes for each receptor group. All experiments were conducted on at least two batches of cRNA synthesis and three oocyte isolations. For each set of experiments, the number of experimental replicates is indicated by large N followed by the number of individual oocytes, represented by small n throughout the manuscript. Data were statistically analyzed using a two-way ANOVA and Tukey’s multiple comparison test to evaluate the means of three or more groups and chaperone treatment using GraphPad Prism 10.4.1.

### 4.7. Transfections

When used, Human Embryonic Kidney cells (HEK293 or HEK293T cells) were seeded in 6-well plates at 10^6^ cells/well. Cells grew overnight prior to transfection. Plasmid DNA samples were diluted using Gibco Opti-MEM medium (Thermo Fisher, Waltham MA, USA) to a ratio of 2 µL Roche X-tremeGENE HP DNA transfection reagent (MilliporeSigma, Burlington MA, USA) for every 1 µg of DNA and then incubated for three days following transfection. Separate transfections of red fluorescent protein (RFP) or green fluorescent protein (GFP) cloned into the Invitrogen pREP4 plasmid served to evaluate transfection efficiency by fluorescence microscopy. Transfections in binding assays were done similarly in 24-well plates seeded at 2 × 10^5^ cells/well using transfection reagents in the same proportions/well but corrected for volume. When mixtures of DNA were used, half the DNA was chrna7 or similar constructs, and the other half was either mixtures of chaperone DNAs or RFP (used as a transfection control in ^125^I-BGT binding assays) or GFP for fluorescent toxin binding assays. Bosc23 cells were transfected similarly, but the ratio of X-tremeGENE to DNA was 3 µL transfectant/µg DNA and GFP was used as the fluorescent protein control.

### 4.8. Binding Assays

Radioactive binding assays were performed to detect surface α7-nAChR or other construct expression as previously described [49]. HEK293 cells were plated at 2 × 10^5^ cells/well in a 24-well plate on day 1 and transfected on day 2. ^125^I-BGT binding assays were performed when cells were 80% confluent or after four days. ^125^I-BGT was made as previously reported [50] with specific activities ranging around 400 Ci/mMole. Binding to Bosc23 cells was determined similarly in 48-well plates with a cell density of 150K/well in 0.3 mL media. Cells were incubated with 0.1 mL 10 nM 125I-BGT (unless stated otherwise) for 3 h in Hanks Buffered Saline (HBSS S, MilliporeSigma, Burlington MA, USA) with 0.1% bovine serum albumin (BSA, Thermo Fisher, Waltham MA, USA) at 4 °C to measure total surface binding. Nonspecific binding was determined by the addition of 1 µM BGT (Thermo Fisher, Waltham MA, USA).

After washing the cells three times in HBSS + 0.1% BSA to remove unbound toxin, cells were lysed for 10–15 min on ice by the addition of 100 µL extraction buffer (0.5 M NaOH, 1% Triton X-100). Lysates were transferred into polypropylene tubes and counted for 1 min with a Packard Cobra gamma counter. Specific binding was determined as the mean of quadruplicate samples of total binding minus the mean of quadruplicate nonspecific binding. The associated errors represent the square root of the sum of the standard deviations for total and nonspecific binding squared.

### 4.9. Fluorescent Binding Assays

Alexa Fluor 647 (Excitation = 650 nm, Emission = 668 nm)-labeled F-BGT (Thermo Fisher, Waltham, MA, USA, B35450) was diluted in phosphate-buffered saline (PBS, Cytiva Hyclone, Logan UT, USA) + 0.1% BSA to make different solutions of F-BGT. 200 µL of 20 nM F-BGT solutions were added to the cells (1.5 × 10^5^/well) in each well and incubated at 37° C for 1 h, then washed 3× with either medium or PBS + 0.1% BSA and imaged on a Keyence fluorescent microscope (Keyence Corporation of America, Itasca, IL, USA).

### 4.10. Fluorescent Images Analysis Using ImageJ

For fluorescent toxin dilution and intensity studies, the images were quadruplicate with one image of each well using a 4× objective. All images were saved in TIFF format and analyzed using NIH ImageJ (versions 1.50-1.53a) as follows: Brightfield images without fluorescence were opened, and four to five regions of interest (ROI) with and without cells were selected. For the images taken on the Cy5 red channel for each ROI, we determined the area of selection in pixels, average pixel intensity within the selection, and the minimum and maximum displayed pixel values. Based on the mean fluorescence intensity per pixel, we first calculated the background in areas without cells. Then we found the mean surface F-BGT fluorescence/pixel after subtracting background and then plotted the corresponding bar graphs representing the cellular expression of different chimeras. In later experiments, an ImageJ macro performed these steps automatically (Supplementary Material SA). Analysis was not blinded but performed on brightfield images without access to fluorescent images to prevent selection bias. A second macro was used to analyze co-transfection (Supplementary Material SB) preformed using a 20× objective.

ImageJ fluorescence were was normalized using the equation Z_i_ = (X_i_ − min(_x_))/(max(_x_) − min(_x_)) × 100 (Z_i_: normalized surface fluorescence intensity of the ith ROI in the dataset; X_i_: surface fluorescence intensity of the ith ROI in the dataset; min(_x_): average surface fluorescence intensity of negative controls (α7 or α7-T267-5HTM4) transfected with GFP (1:1) in each experiment; max(_x_): average surface fluorescence intensity of positive controls (5HT3 chimera) with the combination of NACHO and RIC3 in each experiment).

All results were presented as mean ± SD. One-way ANOVA tests from GraphPad Prism 10.5 software (Carlsbad, CA, USA) were used with post-hoc analysis to compare different groups. If the variances within a group failed the GraphPad normalcy test, the Brown–Forsythe and One-Way ANOVA was used with the Welch post-hoc test. Differences were considered significant at the * *p* < 0.05, ** *p* < 0.01, *** *p* < 0.001, and **** *p* < 0.0001 levels.

## 5. Conclusions

(1) A 5HT3 chimera consisting of the α7 N-terminal (to α7 V224 including the signal sequence), 5HT3 TMs 1-3, α7 ICD (α7 H319 to D468), and 5HT3 M4 + tail does not need additional chaperones to express in HEK293 or Bosc23 cells, unlike α7-nAChR (Figure 1 and Figure 2), corroborating previous reports by Gee et al. [13].
(2) Our data are consistent with Gee et al. [13] that mixing M4 and TMs1-3 from 5HT3 and α7-nAChRs does not allow assembly in the absence of chaperones (Figure 1 and Figure 2).
(3) In the 5HT3 chimera, substituting the 5HT3 tail (SIWHYS) with an α7 nAChR proline plus latch sequence (PNFVEAVSKDFA) allows some expression without the need for RIC3 or NACHO, but adding chaperones does not significantly change expression. This 5HT3-5HT-α7tail construct shows reduced cell surface expression in HEK293 and Bosc23 cells (relative to 5HT-chimera, Figure 1 and Figure 2) and function in oocytes (Figure 3). In oocytes it forms a functional receptor with current similar in amplitude of α7 nAChR (without chaperones). However, in the case when M4 is 5HT3 and TMs1-3 are α7, the resulting α7-5HT-α7tail construct does not express in HEK293 or Bosc23 cells (Figure 1 and Figure 2) and chaperones cannot rescue the expression (Figure 2). However, swapping 5HT3 residues for α7 to restore the SAP motif blocked expression and this could not be reversed with chaperones (Figure 4).
(4) Our results corroborate Kweon et al. [20] that α7-T267-5HTM4 expresses with the help of NACHO and RIC3 but cast doubt on whether L264 and G265 are directly involved in NACHO action (Figure 5). A comparison of the ColabFold-AF2 predictions (Supplementary Figure S7) of α7-T267-5HTM4 (which requires NACHO for surface expression) and 5HT-5HT-α7tail (which does not but expresses (barely) in HEK and Bosc23 cells as well as in oocytes) suggests that the M1 sequence (α7 vs. 5HT3) or possibly the bottom half of M2 specifies whether NACHO is required for assembly. The same AlphaFold predictions imply that L264 and G265 are not located where they can easily interact with NACHO (Supplementary Figure S7), which is corroborated by the model of Hooda et al. [33] (Supplementary Figure S8).
(5) We predicted that substituting an α7 M4 for 5HT3 in T267 constructs should not express, but instead we found that this construct does express with NACHO in the presence of RIC3 (Figure 6). Again, this suggests the bottom half of M2 specifies whether NACHO must be present for expression.

## Figures and Tables

**Table 1:** Constructs tested for effects of M4 mutations on receptor expression.

Construct Name	M1–M3	M4—Latch/Tail Sequence
5HT-chimera	5HT3	DRLLFRIYLLAVLAYSITLVTLWSIWHYS
5HT-C5HT-5HTtail	5HT3	DRLLF**S**IYLVAVIIYSITLVMLWSIWHYS
5HT-L5HT-5HTtail	5HT3	DRLLLMAYSVAVIIYTIGLLMLWSIWHYS
5HT-5HT-α7tail	5HT3	DRLLFRIYLLAVLAYSITLVTLWPNFVEAVSKDFA
5HT-5HT-α7Atail	5HT3	DRLLFRIYLLAVLAYSITLVTLAPNFVEAVSKDFA
5HT-5HT-α7Stail	5HT3	DRLLFRIYLLAVLAYSITLVTSAPNFVEAVSKDFA
5HT-5HT-α7Mtail	5HT3	DRLLFRIYLLAVLAYSITLVMSAPNFVEAVSKDFA
5HT-5HT-α7MtailS	5HT3	DRLLFRIYLLAVLAYSITLVMSA**S**NFVEAVSKDFA
α7-Cα7-5HTtail	α7	DRLLF**S**IYLVAVIIYSITLVMLWSIWHYS
α7-Lα7-5HTtail	α7	DRLLLMAYSVAVIIYTIGLLMLWSIWHYS
α7-5HT-α7tail	α7	DRLLFRIYLLAVLAYSITLVTLWPNFVEAVSKDFA
α7-5HT-α7Atail	α7	DRLLFRIYLLAVLAYSITLVTLAPNFVEAVSKDFA
α7-5HT-α7Stail	α7	DRLLFRIYLLAVLAYSITLVTSAPNFVEAVSKDFA
α7-5HT-α7Mtail	α7	DRLLFRIYLLAVLAYSITLVMSAPNFVEAVSKDFA
α7-5HT-α7MtailS	α7	DRLLFRIYLLAVLAYSITLVMSA**S**NFVEAVSKDFA
α7	α7	DRLCLMAFSVFTIICTIGILMSAPNFVEAVSKDFA

All constructs in Table 1 had an α7-nAChR N-terminal (not designated in the naming scheme), followed by either 5HT3 or α7-nAChR TMs M1–M3 (5HT vs. α7), followed by an α7 ICD, followed by the M4 and tail/latch sequence shown in the table above. For example, the 5HT-C5HT-5HT tail construct has an α7-nAChR N-terminal, 5HT3 M1–M3, the α7 ICD (not designated in the name), and ends with “conservative” (C) mutations in M4 followed by the 5HT3 tail sequence shown in Table 1. Letters in Red denote amino acids unique to α7-nAChR. Underlined letter **S** denotes a substitution of serine for proline at position 491. This **S** amino acid in green is a mistake (an ATG to AGT transposition causing M to S). Molecular modeling to support these chimeras is explained in the Materials and Methods and Supplemental Figure S1.

**Table 2:** Constructs tested for effects of M2 and M4 mutations on T267 chimeras.

Construct Name	M4 & Tail
5HT-chimera	DRL LFRIYLLAVLAYSITLVTLWSIWHYS
α7-T267-5HTM4	DRL LFRIYLLAVLAYSITLVTLWSIWHYS
α7-T267-α7M4	DRL C LMAFSVFTIICTIGILMSAPNFVEAVSKDFA
α7-T267-LYAYL	DRL L LMA Y SV A T II Y TIG L LMSAPNFVEAVSKDFA
α7-T267-LYAL	DRL L LMA Y SV A T II C TIG L LMSAPNFVEAVSKDFA
α7-T267-LYAY	DRL L LMA Y SV A T II Y TIG I LMSAPNFVEAVSKDFA
α7-T267-LYYL	DRL L LMA Y SV F T II Y TIG L LMSAPNFVEAVSKDFA
α7-T267-YAYL	DRL C LMA Y SV A T II Y TIG L LMSAPNFVEAVSKDFA
α7-T267-LAYL	DRLLLMAFSVATIIYTIGLLMSAPNFVEAVSKDFA
α7	DRL C LMAFSVFTIICTIGILMSAPNFVEAVSKDFA
a7-T267-5HT3 G265A	DRL LFRIYLLAVLAYSITLVTLWSIWHYS
a7-T267-5HT3 L264A	DRL LFRIYLLAVLAYSITLVTLWSIWHYS
a7-T267-5HT3 G265K	DRL LFRIYLLAVLAYSITLVTLWSIWHYS
a7-T267-5HT3 L264F	DRL LFRIYLLAVLAYSITLVTLWSIWHYS

T267 chimeras are human α7-nAChRs with amino acids 267-318 in M2 replaced with those from the mouse 5HT3 receptor, and with mutations in M4 as shown above. LYAYL refers to the 5HT3 amino acids in M4 predicted to point towards M3 (Supplemental Figure S1C). These amino acids are switched to 5HT3 from α7-nAChR. These amino acids are identical in mouse 5HT3 and human α7-nAChR. These amino acids in α7-nAChR M4 are predicted to project toward M3.

## Data Availability

The original contributions presented in this study are included in the article/Supplementary Material. Further inquiries can be directed to the corresponding author.

## Supplementary Materials

The following supporting information can be downloaded at: https://www.mdpi.com/article/10.3390/molecules30214235/s1, Figure S1: Molecular modeling of M4 for designing the DNA constructs used in this study; Figure S2: Validation of the fluorescent toxin binding assay; Figure S3: Test of co-transfection in BOSC23 cells; Figure S4: Comparison of ColabFold-AF2-generated structures for the MSAP motif of 5HT-5HT-α7MTail and 5HT-5HT-α7Tail; Figure S5: Comparison of ColabFold-AF2-generated structures for the MSAS motif of 5HT-5HT-α7MTailS and 5HT3-Chimera; Figure S6: SAP mutants do not show much surface expression if M1-M3 do not match M4. Figure S7: ColabFold-AF2 predictions for TMs and C-terminal tails of α7-T267-5HT3 and 5HT-5HT-α7tail; Figure S8: Cryo-EM data of NACHO associated with GABAA α1 with superimposed α7; Supplemental Material SA: Script for the MACRO Fluorescence Analysis Across Channels; Supplemental Material SB: Script for Co-transfection Analysis MACRO.
