# Supplementary material for: Effects of Swapping 5HT3 and α7 Residues in Chimeric Receptor Proteins on RIC3 and NACHO Chaperone Actions
Source: Molecules. 2025 Oct 30;30(21):4235. doi: 10.3390/molecules30214235 (PMC12608704; doi:10.3390/molecules30214235)
Supplement: Supplementary file 1 [file molecules-30-04235-s001.zip › molecules-3893530-supplementary.pdf]

Supplemental Materials for "Effects of swapping 5HT3 and α7 nicotinic receptor amino acids in chimeric receptor proteins on RIC3 and NACHO chaperone actions"

Supplemental Figure S1. Molecular modeling of M4 for designing the DNA constructs used in this study.

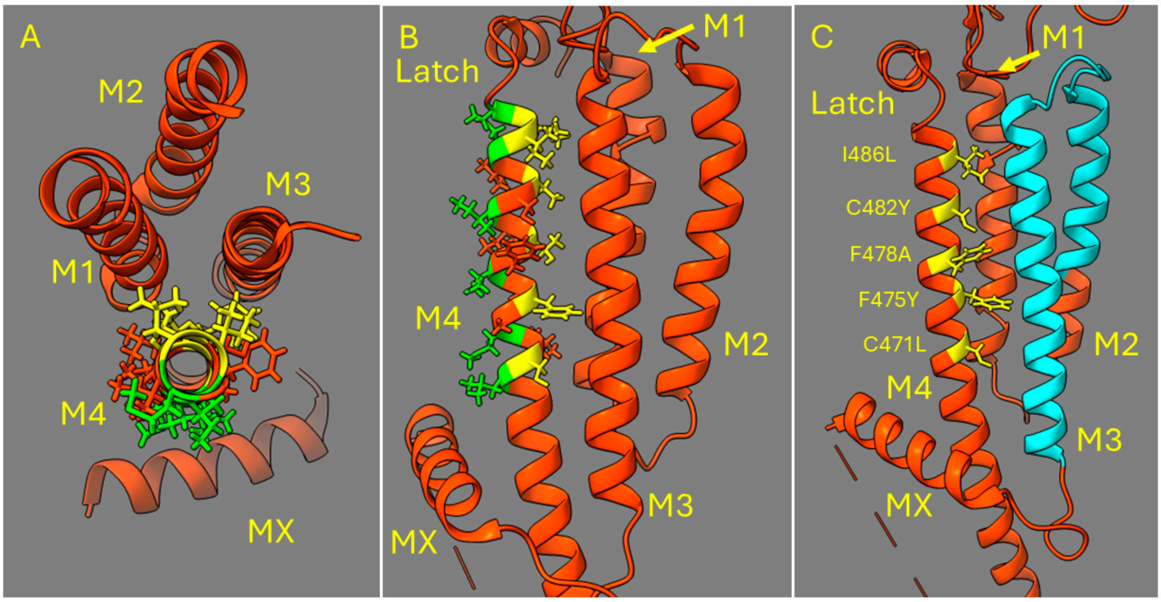

Panel A. The view of α7 M4 from the top. The extracellular N-terminal domain and latch are removed for clarity. Transmembrane domains M1-M4 from a single subunit are shown with the MX helix barely in view. The amino acid residues projecting away from M2 are shown in green, extending into the lipid bilayer, and are considered conservative ("C") substitutions. The yellow amino acids are projecting directly towards M1 or M2. The amino acids that project more laterally are shown in orange-red and are considered less conservative ("L") substitutions. These images are based on PDB 7KOO and rendered in ChimeraX 1.7.

Panel B. A side view of a single subunit colored as in panel A. Now the latch is in view, although the extracellular N-terminal domain is again removed, as is most of the MA helical extension of M4. The MX helix with its loop connecting to M3 is shown with a dotted line at the other end of MX, representing the amino acids in the ICD loop that are not resolvable with cryo-EM.

Panel C. The various mutation sites for the LYAYL series of experiments with the T267 a7-5HT3 chimera are shown. The backbone of the chimera substituted with 5HT3 amino acids is shown in cyan, with the α7 regions shown in orange-red. The individual alpha7 amino acids projecting towards M3 are shown in yellow, along with the corresponding substitution in 5HT3.

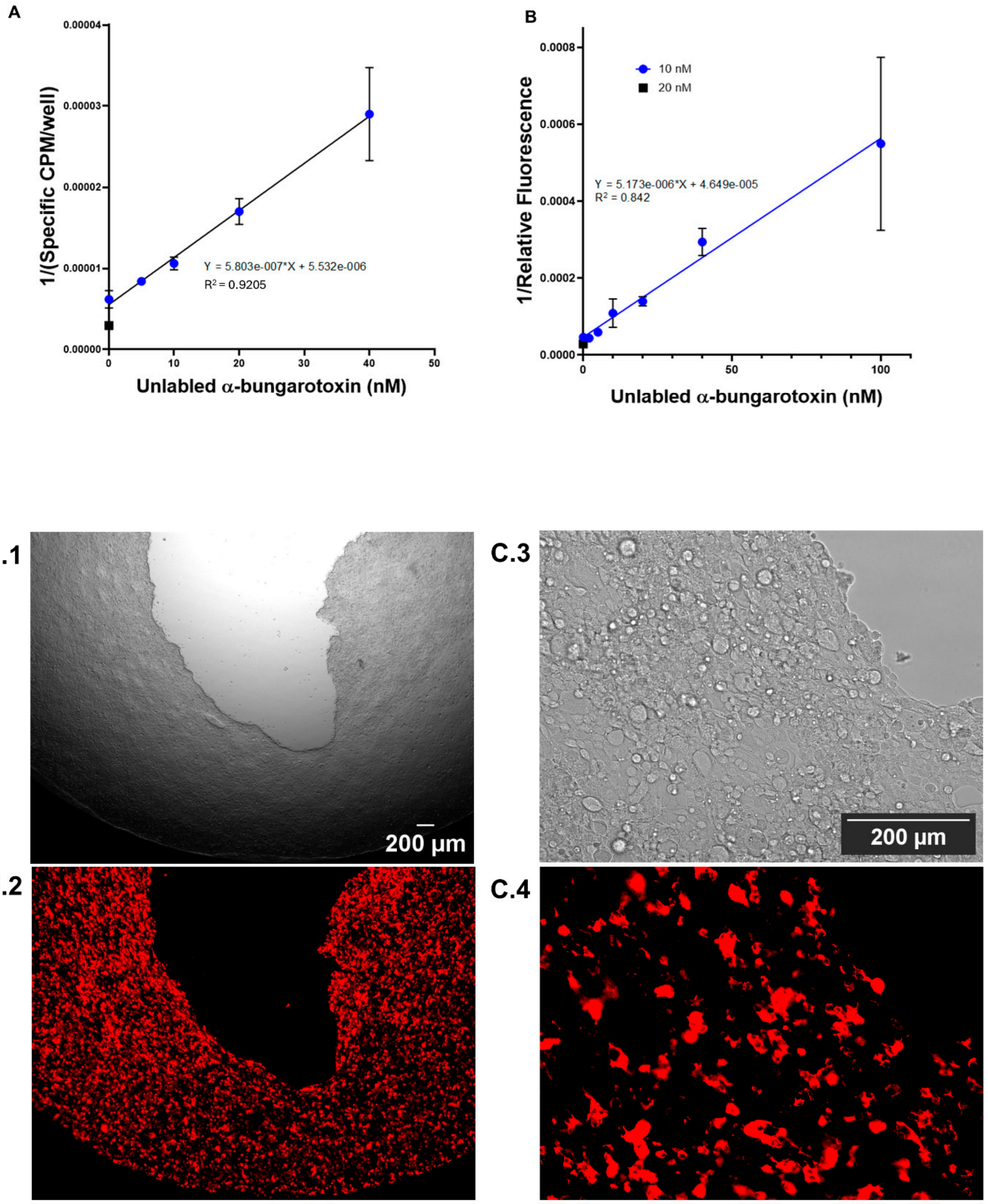

Supplemental Figure S2. Validation of the fluorescent toxin binding assay. A. Binding curve for ^{125}I-BGT binding to HEK cells transfected with 5HT-Chimera, 1/CPM specific binding vs. Concentration of unlabeled BGT. Data are mean $\pm$ SD (1 experiment, quadruplicate samples). The calculated radioactive concentration (-X intercept) is 8.4 nM, close to the nominal concentration of 10 nM. B. Binding curve for F-BGT, 1/normalized fluorescence as a function of

unlabeled BGT toxin using 4x objectives. In panel B, increasing concentrations of unlabled toxin decreased average fluorescence intensity (not shown) in a similar manner to that of radioactively-labeled toxin binding is blocked in panel A. C. Representative brightfield images (C.1 and C.3) using 4x and 20x objectives, respectively of Bosc23 cells transfected with 5HT-Chimera. C.2 and C.4 show the same fields with fluorescence from Alexa Fluor 647 α-bungarotoxin (F-BGT) after the toxin is bound in the absence of unlabeled toxin and then washed.

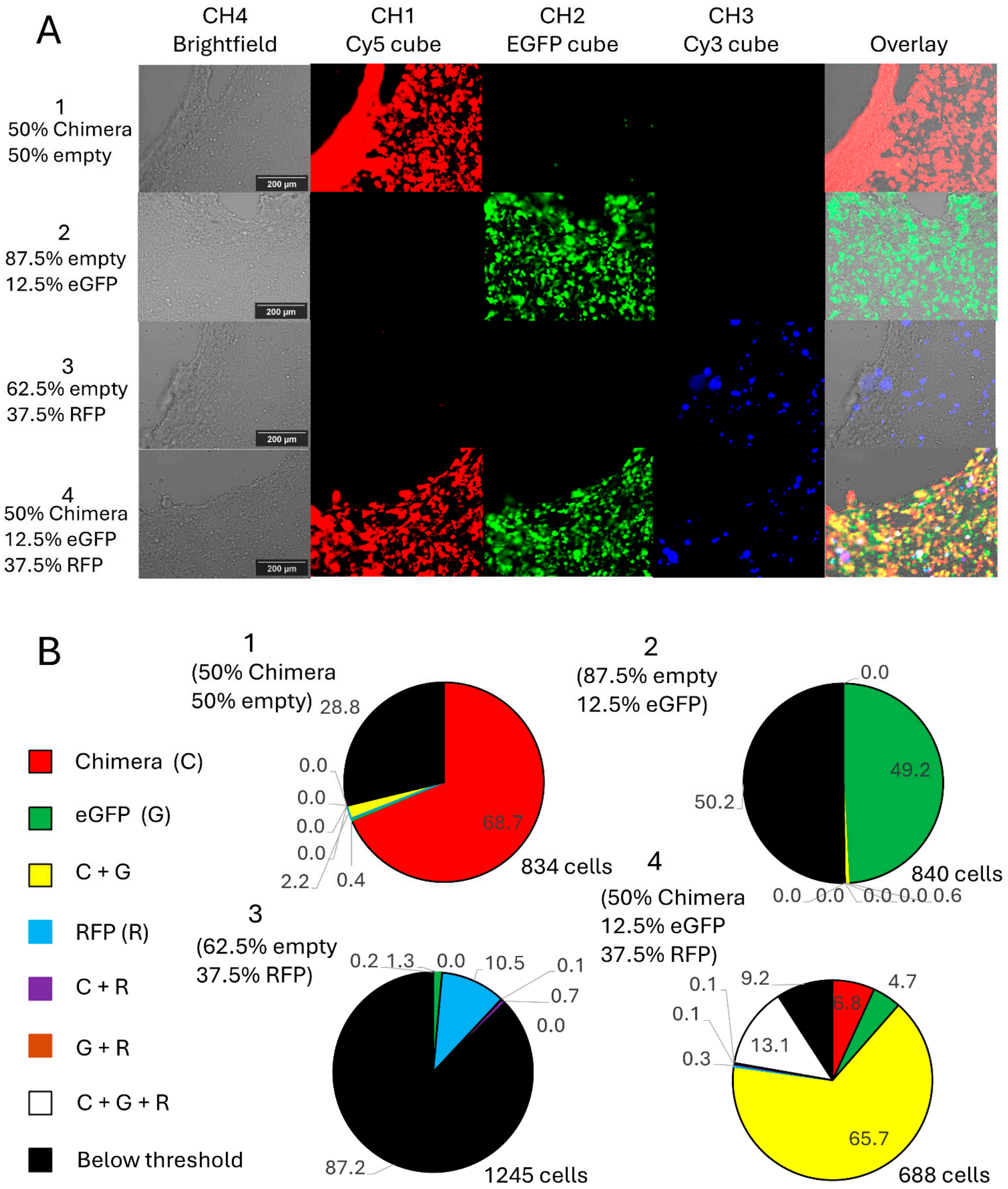

Supplemental Figure S3.Test of co-transfection in BOSC23 cells: BOSC23 cells were plated in a 96-well plate (Fisher catalog 12-566-70) at 50K cells per well in 100 µL medium and transfected the

next day using the ratios seen in the leftmost column of Panel A. Empty = empty pCiNeo plasmid. On day 5, pictures were taken on the Keyence fluorescence microscope with a 20X objective with a Cy5 cube in channel 1, an eGFP cube in channel 2, a Cy3 cube in channel 3 (Channel 3 is artificially set to display blue to distinguish it from channel 1.), and no cube (brightfield) in channel 4. All conditions were treated with 10 nM Alexafluor 647 α-bungarotoxin for 1 hour at 37°C in medium and then washed 3 times with 300 μl medium prior to imaging. Minor crossover fluorescence was observed for condition 1 from 5HT-Chimera into the eGFP channel. In condition 4, which modeled combined transfection of a chimeric receptor with two chaperone genes in a 4:1:3 ratio (in this case, 50% 5HT-Chimera, 12.5% eGFP and 37.5% RFP), 13% of all cells showed evidence of transfection with all three plasmids. The 6th column shows overlays of channels 1-4. Panel B. The analysis shows pie charts of the percentage of cells in each sample that are untransfected (fluorescence not more than 3X background for all three channels), or are only red, green or blue, or are some combination (3X background is sufficient to remove most of the crossover from channel 1 into channel 2). The percentages of total DNA for each construct are shown in parentheses for each analysis. Analysis was performed using Co-transfection Analysis ImageJ macro (Appendix B), with the operator marking the center point of each cell and determining the mean fluorescence in each channel for a 10 pixel x 10 pixel square centered around the point. Marking was performed on the brightfield image with the operator blind to the fluorescent channels, and the position of each mark and square was preserved in a copy of the Region of Interest Manager and an overlay of the locations in each channel. Care was taken to only sample cells in a monolayer. The macro is listed in Appendix B in this supplemental information. In a second experiment, triple transfected cells were 35% of the total cells and in a third, triple transfected cells were 91% of the total cells. Data presented here represents the worst-case scenario.

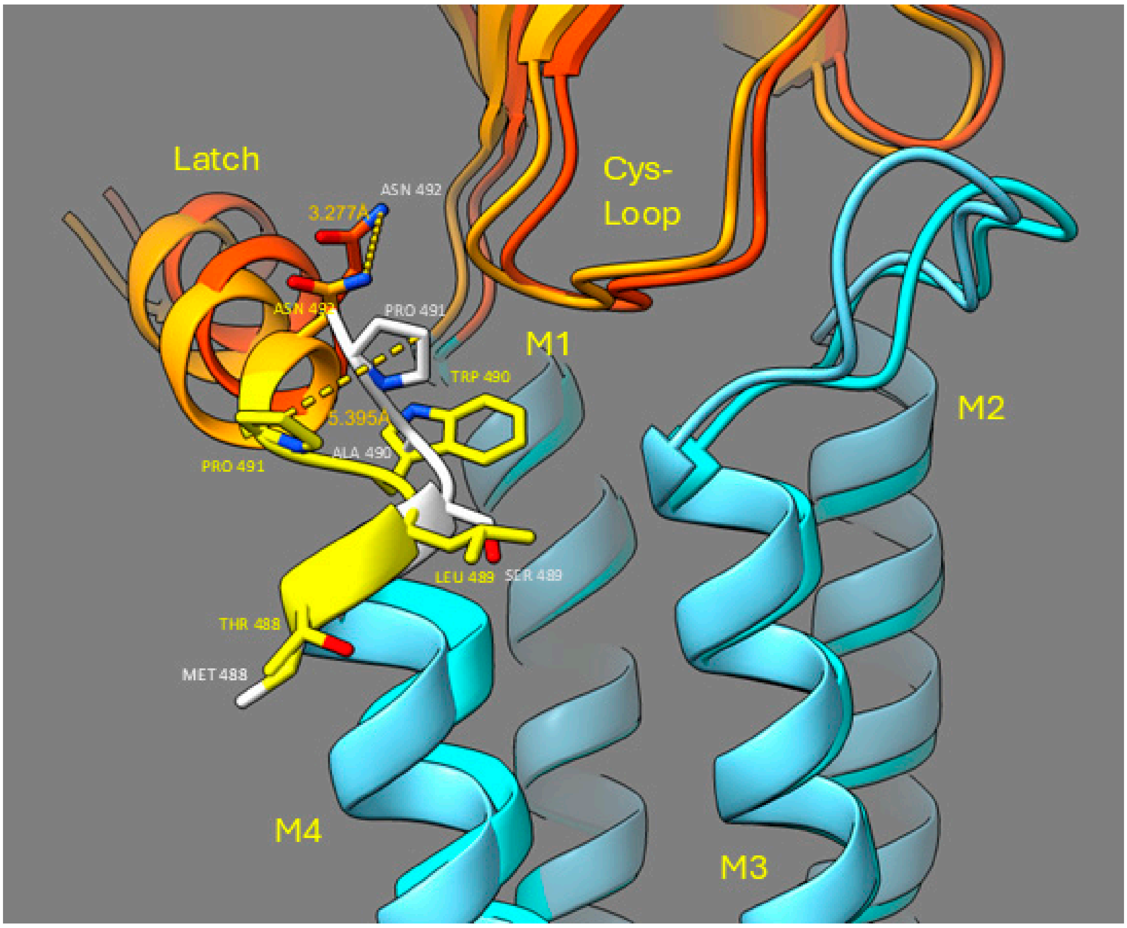

Supplemental Figure S4: Comparison of ColabFold-AF2-generated structures for the MSAP motif of 5HT-5HT-α7MTail (which does not allow surface toxin binding in Bosc23 cells -Figure 4) and the corresponding TLWP sequence of 5HT-5HT-α7Tail (which does [Figure 2] and also allows small currents in oocytes [Figure 3]). The two constructs are otherwise identical. The α7 nAChR backbone of 5HT-5HT-α7MTail is shown in red, the 5HT3 portions in cyan with the SAP motif in white. The α7 backbone regions of 5HT-5HT-α7tail are shown in orange-yellow, the 5HT3 portions in cornflower blue and the TLWP sequence in yellow. P491 is displaced in the two models with a distance >5.3 angstroms between them, while the carboxamide nitrogen of N492, which is common to both models, is displaced less than 3.2 angstroms. The bulky tryptophan 490 in 5HT-5HT-α7tail projects into the cavity between M1 and M3 (A topside view is seen in Supplemental Figure S7). M3 is to the right and M4 to the left with M1 and M2 barely visible to the back. Models were aligned by Matchmaker (RMSD between 352 pruned atom pairs is 0.906 angstroms; across all 393 pairs: 1.358) and rendered with ChimeraX 1.8.

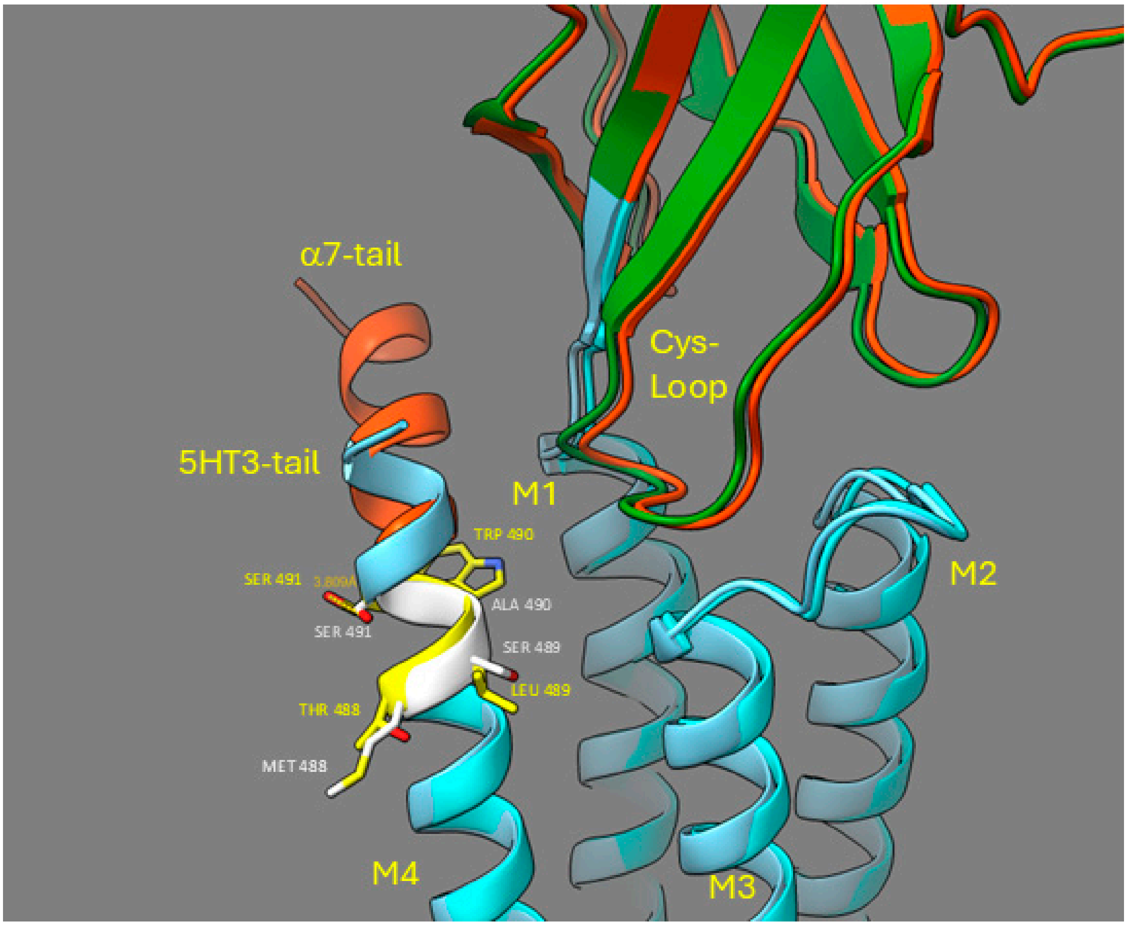

Supplemental Figure S5: Comparison of ColabFold-AF2-generated structures for the MSAS motif of 5HT-5HT-α7MTailS (which does not allow surface toxin binding [Figure 4]) and the TLWS motif of 5HT3-Chimera (which does, see figures 1-6). Both constructs have a serine at position 491, but the 5HT3-Chimera has a C-terminal sequence of IWHYS (5HT3-tail) while 5HT-5HT-α7MTailS has a sequence of NFVEAVSKDFA (α7-tail) following S491. Besides these differences in the tail region and the 3 amino acids before S491, the two constructs are otherwise identical. The α7 nAChR backbone regions of 5HT3-Chimera are shown in green, the 5HT3 portions in light blue, and the TLWS sequence in yellow. The α7 backbone regions of 5HT-5HT-α7MTailS are shown in orange-red, the 5HT3 portions in cyan, and the MSAS sequence in white. In this case, W490 in 5HT3-Chimera projects directly at M1. The helix of the α7-tail is bent relative to the short helix of the 5HT3-tail. M3 is to the right and M4 to the left, with M1 and M2 barely visible to the back. Models were aligned by Matchmaker (RMSD between 383 pruned atom pairs is 0.561 angstroms; across all 388 pairs: 0.704) and rendered with ChimeraX 1.8.

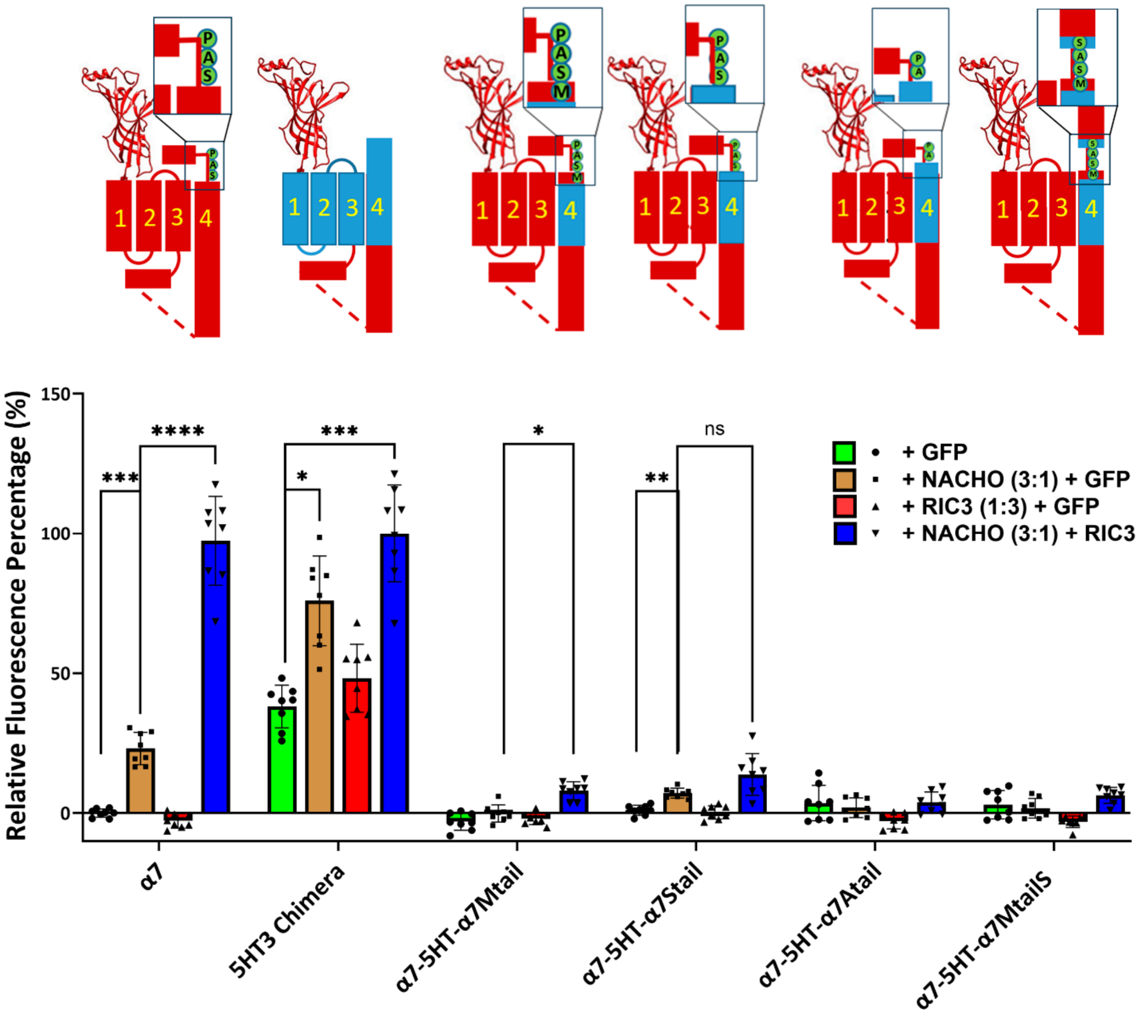

Supplemental Figure S6. SAP mutants do not show much surface expression if M1-M3 do not match M4. These constructs are complementary to Figure 4 but have α7 M1-M3, instead of 5HT3 M1-M3, with 5HT3 M4 and the various mutations to the SAP motif and α7-tail. α7-5HT-α7-Mtail and α7-5HT-α7-Stail show some very slight surface expression in the presence of chaperones. Letters in green circles in the cartoons are as those in Figure 4. N= 2 experiments, quadruplicate samples in each, Brown-Forsythe and Welch ANOVA. * $p < 0.05$, ** $p < 0.01$, *** $p < 0.001$, and **** $p < 0.0001$, ns = not significant.

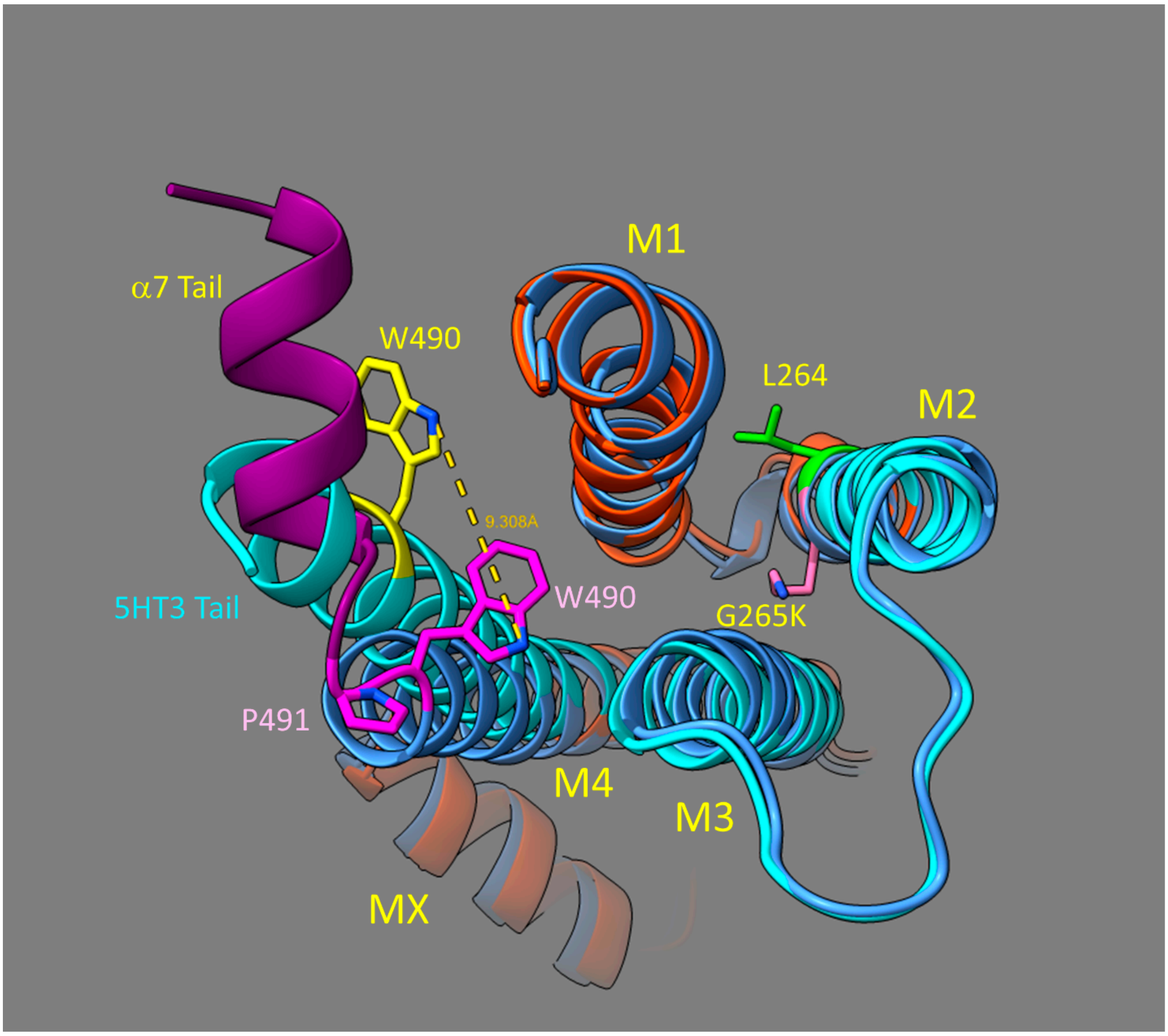

Supplemental Figure S7. ColabFold-AF2 predictions for TMs and C-terminal tails of α7-T267-5HT3 and 5HT-5HT-α7tail constructs looking from the extracellular space (with the N terminals removed). The α7 backbone regions of α7-T267-5HT3 are rendered in orange-red, the 5HT3 regions are cyan with W490 shown in yellow. All the 5HT-5HT-α7tail construct is shown in cornflower blue except W490/P491 in magenta and the α7 tail (NFVEAVSKDFA) in purple. The indole nitrogen in W490 is displaced by 9.3 angstroms between the two models. L264 in M2 of α7-T267-5HT3 is in lime green, and a G265K mutation is in hot pink. The MX helix and the M1-M2 intracellular loop are barely visible in the background. L264F allows surface toxin binding but loses RIC3 enhancement of NACHO, while G265K does not allow expression with chaperones, but G265A expression is unaffected (Figure 5). Aside from the differences shown

here, the rest of these two constructs are identical. Both allow surface toxin binding (without the G265K mutation in α7-T267-5HT3) in BOSC23 cells, but α7-T267-5HT3 requires NACHO (and expresses better with NACHO + RIC3, Figure 6), while the chaperones do not affect the expression of 5HT-5HT-α7tail (Surface expression in Figures 1 and 2 and NACHO slightly inhibits its functional expression in oocytes, Figure 3). The models were superimposed using Matchmaker (RMSD between 372 pruned atom pairs is 0.559 angstroms; across all 387 pairs: 1.025) and rendered with ChimeraX. The G265K mutation was generated from the ColabFold-AF2 prediction using the ChimeraX swapaa command with default rotamers in order to demonstrate the location of this residue.

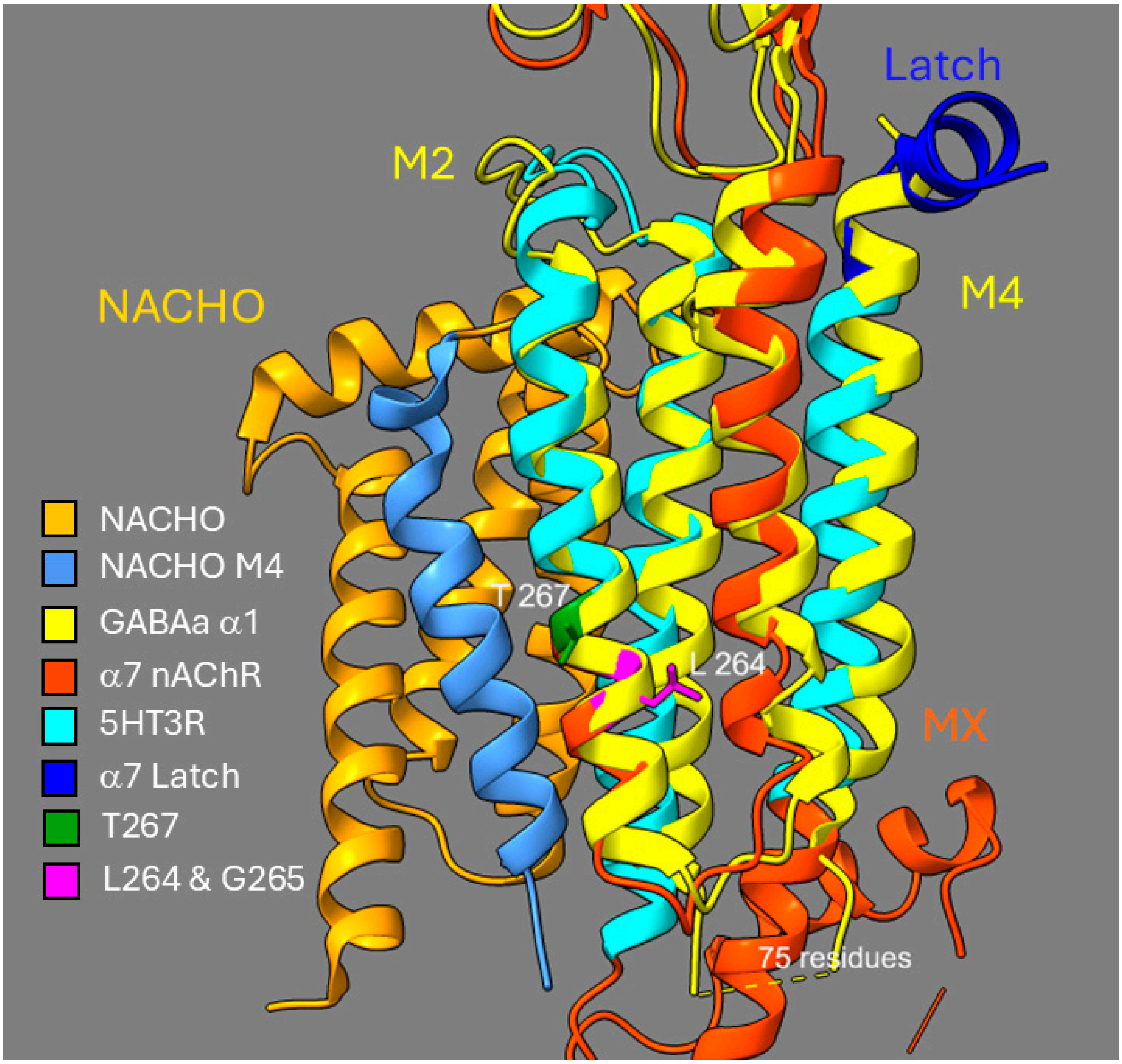

Supplemental Figure S8. Cryo-EM data of NACHO (Tan) associated with $GABA_A$ α1 (Yellow) with superimposed α7 (Orange-Red and Cyan). PDB 9H9E is a dimer of GABAa α1 with two molecules of NACHO (Hooda et al., 2024). Using ChimeraX 10, one GABAa α1 subunit and one molecule of NACHO were removed and one subunit of α7 nAChR (PDB 7EKI) was superimposed on the remaining GABAa α1 subunit using MatchMaker (sequence alignment score = 381.5, RMSD between 80 pruned atom pairs is 1.258 angstroms; [across all 314 pairs: 13.055]). M4 in NACHO (Cornflower blue) is closely associated with α7 M2. T267 (Forest Green) is the transition between α7 and 5HT3 in α7-T267-5HTM4 and α7-T267-α7M4, lying between α7 (Orange-red) and the regions that would be 5HT3 (Cyan except for the Latch colored Blue) in a

T267 α7-5HT3 construct. Note that L264 and G265 (Magenta) are located on α7 M2 facing away from NACHO M4.

Supplemental Material SA: Script for the MACRO Fluoresence Analysis Across Channels saved as an ImageJ file (.ijm).

// Fluorescence Analysis Across Channels MACRO - Written by Vaishali Jain & Ralph Loring
// This script processes multi-channel TIFF images, specifically CH1 (Red), CH2 (Green), and CH3 (Blue) ImageJ channels. It allows users to:
// 1. Manually input Regions of Interest (ROIs) for background and sample (cell) areas on brightfield or phase images.
// 2. Set the scale of the images to pixels and perform measurements (area in pixels, mean gray scale values, standard deviation, Minumum and Maximum values per pixel in each ROI)
// 3. Measure the ROIs selected and adds their labels in each channel so one can verify from visually created images.
// 4. Process Red, Green, and Blue channel one by one: Open TIFF files correspondingly, unstack into particular color channels, and measure the ROIs.
// 5. Save the images modified with ROI(s) and Measurements as .tif files in the original image folder.
// 6. Paste the results for export into an Excel spreadsheet.
// 7. Clear results and prepare for processing the next image (requires re-running the macro).
// 8. Take fluorescent channel measurements blindly starting with brightfield images without being biased by observing any fluorescent images.
//Note that the colors are assigned by ImageJ to stacks for CH1, CH2 and CH3 respectively and can be any color fluorescence.

// Instructions for the user:
// 1. Open the Channel 4 (CH4.tif) image in ImageJ, then click "OK" to start. The file folder should include CH4 and all other channel.tif files to be analyzed.
// 2. Input 5 ROIs (recommended) over background areas (no cells) followed by 't', then 5 ROIs over cells followed by 't', and click "OK".
//   We use rectangles that sample significant regions of the image.
// 3. The script will ask you to paste the result from the "Results" window into Excel after each channel's result comes.
// 4. The macro will automatically process the red, green, and blue ImageJ channels and save the labeled ROIs for future reference in a zipped file.
// 5. The macro checks to see if the .tif images (ending in CH1.tif, CH2.tif, and CH3.tif) are present in the image directory before analysing each channel.
// 6. If a channel is not present, the macro will inform the user before proceeding with the rest. Click OK to proceed.
// 7. Paste the data into an open Excel spreadsheet when prompted. The data is preloaded into the clipboard so no copying is required.
// 8. Re-run the macro to analyze the next images in a different folder.
// 9. The macro will handle as many background and sample (cell) ROIs as are input, but a minimum of 5 each is recommended to get good sampling of the image.
//10. It's up to the user to keep track of which ROI is which (background vs. sample) in the data.
//11. The user will see "Red", "Blue" and "Green" "Channel_ROI.tif" files stored in the image folder with the ROIs labeled and preserved in the image file as they are analyzed.
//12. The mean fluorescence of that image is calculated as the difference between the mean density of samples minus the mean density of background ROIs in the Excel data.
//13. Data from multiple images under identical conditions should be combined (usually quadruplicate images).

```
// Get directory path
waitForUser("Open CH4 image in ImageJ, then click \"OK\".");
path = getDirectory("image");

// Get list of files in directory
list = getFileList(path);
// Note, CH4.tif is already open and then opening again causes an additional file to be created that's not
used
// and needs to be closed again ?? Is this true and if so, where does this happen?

// Setting measurements
run("Set Measurements...", "area mean standard min display redirect=None decimal=1");
// Setting scale to pixels
run("Set Scale...", "distance=0 known=0 pixel=1 unit=pixel");

// Input ROIs manually
waitForUser("Enter 5 background ROIs (No cells) followed by t, then 5 cell ROIs followed by t, then click
\"OK\".");


// Defining an empty array for ROIs
array = newArray(0);
// Save ROIs in Zip file
roiManager("Save", path + "RoiSet.zip");

// Checking and processing CH1 (Red) channel if it exists, otherwise skip the analysis.

foundCH1 = false;
for (i = 0; i < list.length; i++) {
   if (endsWith(list[i], "CH1.tif")) {
      open(path + list[i]);
      foundCH1 = true;
      break;
   }
}

// If CH1 exists, convert scale to pixels, unstack image and open Red channel
if (foundCH1) {
   run("Set Scale...", "distance=0 known=0 pixel=1 unit=pixel");
   run("Stack to Images");
   selectWindow("Green");
   close();
   selectWindow("Blue");
   close();
   selectWindow("Red");
   run("16-bit");
```

```
    selectWindow("Red");
    count = roiManager("count");
    array = newArray(count);
    for (i = 0; i < array.length; i++) {
        array[i] = i;
    }
    roiManager("select", array);
    roiManager("Show All");
    roiManager("Measure");

    roiManager("select", array);
    roiManager("Show All with labels");
    run("Flatten");
    saveAs("tiff", path + "RedChannel_ROIs.tif");

    selectWindow("Results");
    String.copyResults();
    waitForUser("Copy CH1 Results to Excel, then click \"OK\".");
    run("Clear Results");
} else {
    waitForUser("CH1 not found, skipping Red channel analysis and proceeding to the next channel-click
\"OK\".");
}

// Checking and processing  CH2 (Green) channel if it exists, otherwise skip the analysis.

foundCH2 = false;
for (i = 0; i < list.length; i++) {
    if (endsWith(list[i], "CH2.tif")) {
        open(path + list[i]);
        foundCH2 = true;
        break;
    }
}

if (foundCH2) {
    run("Set Scale...", "distance=0 known=0 pixel=1 unit=pixel");
    run("Stack to Images");
    selectWindow("Blue");
    close();
    selectWindow("Red");
    close();
    selectWindow("Green");
    run("16-bit");

    roiManager("select", array);
    roiManager("Show All");
    roiManager("Measure");
```

```
    roiManager("select", array);
    roiManager("Show All with labels");
    run("Flatten");
    saveAs("tiff", path + "GreenChannel_ROIs.tif");

    selectWindow("Results");
    String.copyResults();
    waitForUser("Copy CH2 Results to Excel, then click \"OK\".");
    run("Clear Results");
} else {
    waitForUser("CH2 not found, skipping Green channel analysis and proceeding to the next channel-
click \"OK\".");
}

// Checking and processing CH3 (Blue) channel if it exists, otherwise skip analysis and deselect all the
tabs.

foundCH3 = false;
for (i = 0; i < list.length; i++) {
   if (endsWith(list[i], "CH3.tif")) {
     open(path + list[i]);
     foundCH3 = true;
     break;
   }
}

if (foundCH3) {
    run("Set Scale...", "distance=0 known=0 pixel=1 unit=pixel");
    run("Stack to Images");
    selectWindow("Red");
    close();
    selectWindow("Green");
    close();
    selectWindow("Blue");
    run("16-bit");

    roiManager("select", array);
    roiManager("Show All");
    roiManager("Measure");

    roiManager("select", array);
    roiManager("Show All with labels");
    run("Flatten");
    saveAs("tiff", path + "BlueChannel_ROIs.tif");

    selectWindow("Results");
    String.copyResults();
```

```
    waitForUser("Copy Blue Channel Results to Excel, then click \"OK\".");
} else {
    waitForUser("CH3 not found, skipping Blue channel analysis and proceeding to the next channel-click
\"OK\".");
}

// Erases ROI manager and closes windows to be ready for next image
roiManager("Deselect");
roiManager("Delete");
selectWindow("Results");
close("Results");
selectWindow("ROI Manager");
close("ROI Manager");


// Close all windows to prepare for the next analysis
run("Close All");
```

Supplemental Material SB: Script for Co-transfection Analysis MACRO (saved as an ImageJ .ijm file)

```
// Macro Co-Transfection Analysis - Written by Vaishali Jain & Ralph Loring
// Description:
// 1. This macro helps assess the extent of co-transfection in fluorescence microscopy images by sampling 10x10 pixel square ROIs across multiple channels (Red, Green, Blue) based on user-selected cell locations.
// 2. User opens CH4 (brightfield) image from a folder containing 1_CH1.tif, 1_CH2.tif, 1_CH3.tif, and 1_CH4.tif.
// 3. User selects 10 background ROIs (no cells) followed by about 100 cell ROIs (clicking on center of each cell).
// 4. The macro creates 10x10 pixel ROIs at each clicked point.
// 5. After pressing the spacebar, the macro automatically measures mean intensity for all selected ROIs across CH1 (Red), CH2 (Green), and CH3 (Blue) channels. It also checks whether those channels exist.
// 6. Results are copied to clipboard and ROIs are saved as a zipped file
// 7. All input images should be in the same directory and named as 1_CH1.tif (Red), 1_CH2.tif (Green), 1_CH3.tif (Blue), and 1_CH4.tif (Brightfield).
// 8. ROI measurements can be pasted into Excel for downstream analysis.
// 9. Designed for 20X objective magnification on a Keyence X700.

// OUTPUT:
//1. ROIs saved in zipped folder for later inspection
// 2. Results copied to clipboard

// INSTRUCTIONS FOR USERS:

// This macro samples 10x10 pixel square ROIs to analyze co-transfection across multiple channels.

// Please follow these steps when running the macro:

// 1. Ensure your working folder contains the following TIFF files:
//    1_CH1.tif   Red fluorescence (optional)
//    1_CH2.tif   Green fluorescence (optional)
//    1_CH3.tif   Blue fluorescence (optional, but at least one fluorescence image is required, and less than two makes little sense)
//    1_CH4.tif   Brightfield (used for selecting ROIs)

// 2. When prompted, open the CH4 (brightfield) image in ImageJ by pushing it onto the ImageJ ribbon.

// 3. The ROI Manager will open automatically.

// 4. Using your mouse:
//    - First, click on **10 background regions** (areas without cells).
//    - Then, click on at least **100 cell centers** (each click creates a 10x10 pixel ROI).
// 5. After placing all ROIs (total >= 110), press the **spacebar** to continue.

// 6. The macro will:
//    - Measure fluorescence intensities across CH1 (Red), CH2 (Green), and CH3 (Blue).
```

//   - Save your selected ROIs (in a zipped file) as well as an image with ROIs superimposed on each channel
//   - Copy the measurement results to your clipboard.

// 7. The user pastes the copied results into Excel for downstream analysis.
//Note: This MACRO runs most of the time, but occasionally will throw exceptions and has even crashed.
//The cause of this instability has not been identified, but the data generated when it does run (over 95% of the time) is valid.

// CODE:

```
// Get directory path and list of files
waitForUser("Open CH4 image in ImageJ, then click 'OK' to begin.");
path = getDirectory("image");
list = getFileList(path);

// Set measurement settings and scale to pixels
run("Set Measurements...", "mean standard min display redirect=None decimal=1");
run("Set Scale...", "distance=0 known=0 pixel=1 unit=pixel");

waitForUser("After clicking 'OK', the ROI Manager will open.");

// Clear key presses and show ROI Manager
setKeyDown("none");
roiManager("Reset");
roiManager("Show All with labels");

waitForUser("Click 'OK' and select 10 background ROIs and 100 cell ROIs using your mouse.\n\nAfter selecting all ROIs, press the 'spacebar' to end.");

// Initialize mouse click tracking
leftButton = 16;
x2 = -1; y2 = -1; z2 = -1; flags2 = -1;

// Start mouse click loop for ROI placement
while (true) {
    if (isKeyDown("space")) {
        // Stop when space bar is pressed
        break;
    }

    getCursorLoc(x, y, z, flags);

    if (flags != flags2) {
        if (flags > 16) {
            flags = 0;
        }
```

```
    if ((flags & leftButton) != 0) {
      // Create 10x10 ROI at clicked point and add to manager
      makePoint(x, y);
      run("Specify...", "width=10 height=10 x y centered");
      roiManager("Add & Draw");
      flags = 0;
    }
  }

  // Track previous state
  x2 = x; y2 = y; z2 = z; flags2 = flags;
  wait(20);
}

// Initialize and show all ROIs
array = newArray(roiManager("count"));

//Save image of CH4 with ROIs
roiManager("select", array);
roiManager("Show All with labels");
// Save ROIs in Zip file
roiManager("Save", path + "RoiSet.zip");

// Save flattened image with CH4 ROIs
run("Flatten");
saveAs("jpeg", path + "Ch4_ROIs.jpg");

// Analyze CH1 (Red) channel

foundCH1 = false;
for (i = 0; i < list.length; i++) {
  if (endsWith(list[i], "CH1.tif")) {
    open(path + list[i]);
    foundCH1 = true;
    break;
  }
}

if (foundCH1) {
  run("Set Scale...", "distance=0 known=0 pixel=1 unit=pixel");
  run("Stack to Images");

  // Close non-red channels
  selectWindow("Green"); close();
  selectWindow("Blue"); close();
  selectWindow("Red");
  run("16-bit");
```

```
    // Apply ROIs and measure intensities
    count = roiManager("count");
    array = newArray(count);
    for (i = 0; i < count; i++) {
        array[i] = i;
    }

    run("Clear Results");
    roiManager("select", array);
    roiManager("Show All");
    roiManager("Measure");

    // Save ROI overlay on red channel
    roiManager("select", array);
    roiManager("Show All with labels");
    run("Flatten");
    saveAs("jpeg", path + "RedChannel_ROIs.jpg");
    selectWindow("Results");
    String.copyResults();
    waitForUser("Copy CH1 Results to Excel, then click \"OK\".");
    run("Clear Results");

} else {
    waitForUser("CH1 not found, skipping Red channel analysis. Click 'OK' to continue.");
}

// Analyze CH2 (Green) channel

foundCH2 = false;
for (i = 0; i < list.length; i++) {
    if (endsWith(list[i], "CH2.tif")) {
        open(path + list[i]);
        foundCH2 = true;
        break;
    }
}

if (foundCH2) {
    run("Set Scale...", "distance=0 known=0 pixel=1 unit=pixel");
    run("Stack to Images");

    // Close non-green channels
    selectWindow("Blue"); close();
    selectWindow("Red"); close();
    selectWindow("Green");
    run("16-bit");
```

```
    // Apply ROIs and measure intensities
    roiManager("select", array);
    roiManager("Show All");
    roiManager("Measure");

    // Save ROI overlay on green channel
    roiManager("select", array);
    roiManager("Show All with labels");
    run("Flatten");
    saveAs("jpeg", path + "GreenChannel_ROIs.jpg");
    selectWindow("Results");
    String.copyResults();
    waitForUser("Copy CH2 Results to Excel, then click \"OK\".");
    run("Clear Results");
} else {
    waitForUser("CH2 not found, skipping Green channel analysis. Click 'OK' to continue.");
}

// Analyze CH3 (Blue) channel

foundCH3 = false;
for (i = 0; i < list.length; i++) {
    if (endsWith(list[i], "CH3.tif")) {
        open(path + list[i]);
        foundCH3 = true;
        break;
    }
}

if (foundCH3) {
    run("Set Scale...", "distance=0 known=0 pixel=1 unit=pixel");
    run("Stack to Images");

    // Close non-blue channels
    selectWindow("Red"); close();
    selectWindow("Green"); close();
    selectWindow("Blue");
    run("16-bit");

    roiManager("select", array);
    roiManager("Show All");
    roiManager("Measure");

 // Save ROI overlay on blue channel
    roiManager("select", array);
    roiManager("Show All with labels");
    run("Flatten");
    saveAs("jpeg", path + "BlueChannel_ROIs.jpg");
```

```
    // Prompt user to copy results
    selectWindow("Results");
    String.copyResults();
    waitForUser("Copy CH3 Results to Excel, then click 'OK' to proceed.");

} else {
    waitForUser("CH3 not found, skipping Blue channel analysis. Click 'OK' to finish.");
}

// Erases ROI manager and closes windows to be ready for next image
roiManager("Deselect");
roiManager("Delete");
selectWindow("Results");
close("Results");
selectWindow("ROI Manager");
close("ROI Manager");


// Close all windows to prepare for the next analysis
run("Close All");
```
